# Paragangliomas arise through an autonomous vasculo-angio-neurogenic program inhibited by imatinib

**DOI:** 10.1007/s00401-017-1799-2

**Published:** 2018-01-05

**Authors:** Fabio Verginelli, Silvia Perconti, Simone Vespa, Francesca Schiavi, Sampath Chandra Prasad, Paola Lanuti, Alessandro Cama, Lorenzo Tramontana, Diana Liberata Esposito, Simone Guarnieri, Artenca Sheu, Mattia Russel Pantalone, Rosalba Florio, Annalisa Morgano, Cosmo Rossi, Giuseppina Bologna, Marco Marchisio, Andrea D’Argenio, Elisa Taschin, Rosa Visone, Giuseppe Opocher, Angelo Veronese, Carlo T. Paties, Vinagolu K. Rajasekhar, Cecilia Söderberg-Nauclér, Mario Sanna, Lavinia Vittoria Lotti, Renato Mariani-Costantini

**Affiliations:** 1 Laboratory of General Pathology, Center of Aging Science and Translational Medicine (CeSI-MeT), Gabriele d’Annunzio University, Via Luigi Polacchi 11, 66100 Chieti, Italy; 2 Department of Pharmacy, Gabriele d’Annunzio University, Via dei Vestini 31, 66100 Chieti, Italy; 3 Department of Medical, Oral and Biotechnological Sciences, Gabriele d’Annunzio University, Via dei Vestini 31, 66100 Chieti, Italy; 4 Familial Cancer Clinic and Oncoendocrinology, Veneto Institute of Oncology, IRCCS, Padua, Italy; 5 Otology and Skull Base Unit, Gruppo Otologico Piacenza-Roma, Via Antonio Emmanueli, 42, 29121 Piacenza, Italy; 6 Department of Experimental Medicine, Sapienza University of Rome, Viale Regina Elena 324, 00161 Rome, Italy; 7 Department of Oncology-Hematology, Service of Anatomic Pathology, Guglielmo da Saliceto Hospital, Via Taverna 49, 29100 Piacenza, Italy; 8 Department of Surgery, Memorial Sloan Kettering Cancer Center, New York, NY 10065 USA; 9 Department of Medicine, Experimental Cardiovascular Research Unit, and Department of Neurology, Center for Molecular Medicine, Karolinska Institute, Solnavägen 1, 171 77 Solna, Stockholm Sweden

**Keywords:** Paraganglioma, Mesenchymal stem-like cells, Xenograft, Vasculogenesis, Neurogenesis, Mitochondria

## Abstract

Tumours can be viewed as aberrant tissues or organs sustained by tumorigenic stem-like cells that engage into dysregulated histo/organogenetic processes. Paragangliomas, prototypical organoid tumours constituted by dysmorphic variants of the vascular and neural tissues found in normal paraganglia, provide a model to test this hypothesis. To understand the origin of paragangliomas, we built a biobank comprising 77 cases, 18 primary cultures, 4 derived cell lines, 80 patient-derived xenografts and 11 cell-derived xenografts. We comparatively investigated these unique complementary materials using morphofunctional, ultrastructural and flow cytometric assays accompanied by microRNA studies. We found that paragangliomas contain stem-like cells with hybrid mesenchymal/vasculoneural phenotype, stabilized and expanded in the derived cultures. The viability and growth of such cultures depended on the downregulation of the miR-200 and miR-34 families, which allowed high PDGFRA and ZEB1 protein expression levels. Both tumour tissue- and cell culture-derived xenografts recapitulated the vasculoneural paraganglioma structure and arose from mesenchymal-like cells through a fixed developmental sequence. First, vasculoangiogenesis organized the microenvironment, building a perivascular niche which in turn supported neurogenesis. Neuroepithelial differentiation was associated with severe mitochondrial dysfunction, not present in cultured paraganglioma cells, but acquired in vivo during xenograft formation. Vasculogenesis was the Achilles’ heel of xenograft development. In fact, imatinib, that targets endothelial-mural signalling, blocked paraganglioma xenograft formation (11 xenografts from 12 cell transplants in the control group versus 2 out of 10 in the treated group, *P* = 0.0015). Overall our key results were unaffected by the *SDHx* gene carrier status of the patient, characterized for 70 out of 77 cases. In conclusion, we explain the biphasic vasculoneural structure of paragangliomas and identify an early and pharmacologically actionable phase of paraganglioma organization.

**Electronic supplementary material:**

The online version of this article (10.1007/s00401-017-1799-2) contains supplementary material, which is available to authorized users.

## Introduction

The parallelism between some tumours and their organs or tissues of origin suggests that tumourigenesis, like organogenesis, might pass through definable formative stages, orchestrated by stem-like cells whose engagement in complementary, albeit aberrant, differentiation processes could be spatiotemporally connected with the organization of the neoplastic microenvironment [15, 30]. This hypothesis implies that preventive and curative interventions should be designed and targeted based on a thorough understanding of tumour histogenesis.

Paragangliomas (PGLs) are rare hypervascular tumours of the paraganglia, organelles connected with the autonomic branches of the cranial (head and neck) and thoraco-lumbar nerves [47]. They are frequently associated with predisposing mutations in one of several nuclear genes, most relevantly *SDHA/B/C/D*, that encode the four subunits of succinate dehydrogenase (SDH), a mitochondrial (mt) enzyme participating in both oxidative phosphorylation and the Krebs cycle, and *SDHAF2*, whose product is required for SDHA flavination (collectively the *SDHx* genes) [47]. PGLs grow slowly, but are highly infiltrating, may unpredictably metastasize and are refractory to chemo/radiotherapy. Head and neck PGLs (~ 20% of all PGLs) are of particular concern, as they spread along the regional neurovascular structures towards the skull base, may insinuate intracranially and may compress the brainstem [61]. Surgical resection is challenging, and postoperative deficits of the lower cranial nerves are a significant cause of morbidity and permanent disability [4]. The difficult recruitment of patients, the need of long follow-up and the lack of preclinical models are major barriers to the development or repurposing of drugs for PGL treatment [47, 61].

PGLs recapitulate the histostructure of normal paraganglia. The cardinal feature shared by PGLs and paraganglia is the integration of a neurosecretory network, consisting in nests or cords of glia-bound neuroepithelial cells (“zellballens”), with an angiomatous vasculature [7]. The histostructural convergence suggests that paragangliar tumorigenesis exploits a deeply embedded organogenetic program. In this regard stem-like cells have been detected in PGLs [9, 46, 75]. However, the current view, reflected in the WHO classification [71], is that PGLs are of neuroendocrine (i.e., neuroepithelial) origin, while their vasculature, although aberrant, is thought to arise from extrinsic angiogenic ingrowth and is thus relegated to a secondary and subordinate role [40]. This influences the current strategies of PGL prevention and therapy [47, 61].

Here, using *SDHx*-characterized head and neck PGLs and derived in vitro and in vivo models, we demonstrate that the vascular and the neural PGL components develop sequentially from stem-like tumour cells characterized by a hybrid mesenchymal/vasculoneural phenotype. Thus, PGLs are fundamentally vasculoneural tumours and vasculoangiogenesis is the primary and pharmacologically actionable tumorigenic process. Our findings, that support an organogenetic model of paragangliar tumourigenesis, are applicable to PGLs that are both related and unrelated to germline *SDHx* mutations.

## Patients, materials, and methods

### Patients, samples and *SDHx* mutational analysis

The case series (77 PGL patients recruited between November 2009 and June 2017 at Gruppo Otologico, Piacenza, Italy) is listed in Table S1 (Online Resource 1). The patients did not receive radio/chemotherapy but preoperative tumour embolization was routinely performed (except for patients with tympanic PGL) [61]. Case acronyms encode PGL (P) localization (carotid body, C; vagal, V; tympanic, T; tympano-jugular, TJ) followed by progressive number. Solid biospecimens, evaluated fresh to exclude areas damaged by embolization, were differentially sampled within 5 min from excision in: (a) RNAlater (nucleic acids); (b) high-glucose DMEM with penicillin, streptomycin and fungizone (cytofluorimetry, cell culture, ex vivo culture, xenotransplantation, JC-1 assays); (c) liquid nitrogen (biochemical studies); (d) 2% paraformaldehyde (PFA) and 0.2% glutaraldehyde in PBS at 4 °C (8 h), then 2% PFA (ApoTome immunofluorescence, AIF); (e) 2% glutaraldehyde in PBS at 4 °C (light and transmission electron microscopy, TEM). Samples (d)–(e) were trimmed in ~ 3 × 3 mm pieces before fixation. Processing was restricted to (c)–(e) when scarce tissue was available. Anticoagulated blood (20 ml) for mutational analysis and formalin-fixed/paraffin-embedded (FFPE) samples for standard histopathology and immunohistochemistry (IHC) were routinely obtained.

Point mutations and large deletions/rearrangements in the *SDHA*, *SDHB*, *SDHC*, *SDHD* and *SDHAF2* genes and SDHB protein immunostaining were assessed as described [7, 67]. Methods used for miRNAstudies are detailed in the Online Resource 2 (Appendix to Materials and Methods).

### Immunomorphological and ultrastructural studies

AIF, that generates high resolution images in the focal plane by computational optical sectioning [57], was used to investigate the expression and localization of marker proteins in semithin (200 nm-thick) cryo-ultramicrotomy sections of PGL and patient-derived xenograft (PDX) tissues that had been lightly fixed in 2% PFA and 0.2% glutaraldehyde in cold PBS at surgery (see above). Sections were analysed using an ApoTome Axio Observer Z1 inverted microscope (Zeiss) equipped with an AxioCam MRm Rev.3. Colocalizations were assessed with Axio Vision software, release 4.6.3 (Zeiss). AIF was also used to analyse cells, which were grown on CultureSlides (BD Falcon), fixed in 4% PFA for 30 min, washed twice in PBS, stained and directly examined as above. Alternatively, cells were analysed using an LSM 800 confocal laser scanning microscope (Zeiss). Marker proteins and relative antibodies are listed in the Online Resource 2 (Appendix to Materials and Methods). Positive and negative controls were added to all the immunostaining procedures. Negative controls included omission of primary antibody, blocking with secondary antibody and substitution of primary antibody with irrelevant isotype-matched immunoglobulin. Standard IHC on FFPE sections for S100, synaptophysin (SYP), chromogranin A (CGA), vimentin, alpha-smooth muscle actin (SMA), CD34 and NOTCH1 was as reported [7]. For TEM, PGL, PDX and CDX samples (~ 3 mm in maximum diameter) or cell pellets (at least 1 × 10^6^ cells) were processed as described [7] and examined using a Philips CM10 transmission electron microscope. Mitochondrial cross-sectional areas (µm^2^) were determined on electron micrographs using NIH ImageJ and analysed with one-way ANOVA and paired samples *t* test. Methods used for cytofluorimetry and western blotting are detailed in the Online Resource 2.

### Cell cultures

Primary PGL cultures were established from cells dissociated with Accumax (Millipore) according to manufacturer’s protocol and grown at 37 °C under ambient atmosphere plus 5% CO_2_ in DMEM-F12 supplemented with 20–10% FBS (early to late passages), 3.5 mM l-glutamine, 100 IU penicillin and 100 μg/ml streptomycin. Primary cultures were immortalized by retroviral-mediated transduction of full-length hTERT and/or simian virus 40 (SV40) large-tumour (LT) antigen (Addgene, https://www.addgene.org/). The virus was constructed infecting HEK293 cells with a combination of vectors bringing Gag-polymerase, virus envelope proteins and full-length hTERT (pBabe-hygro-hTERT) or SV40LT (pBabe-puro SV40 LT) cDNAs. Mid-confluence cultures at passage 4 were exposed to 0.4 µm filtered supernatants from the retroviral packaging lines for 3–6 h with 5 µg/ml polybrene, and grown for two passages before second infection (when performed). Cells were selected with puromycin and/or hygromycin B until all untransduced cells had been eliminated.

For neurosphere formation primary or immortalized cells were cultured serum-free in CellRepel flasks (Eppendorf) with DMEM:F12 or NeuroBasal media supplemented with N2, B27 (Life Technologies) and growth factors (FGFb, IGF1, EGF, each 20 ng/ml) (Prospec). For the Matrigel assay [19] cells (9 × 10^4^/well) were seeded in triplicate in 35 mm Cell Imaging Dishes (Eppendorf) on top of a layer of growth factor-reduced Matrigel (BD) in DMEM:M199 (1:1) supplemented with 20% FBS, 18U/ml heparine (Clarisco) and 50 μg/ml endothelial cell growth factor (Sigma). The cultures were examined by phase contrast at 1–2–4 h, fixed for 30 min adding 4% PFA in PBS to an equal volume of medium, washed twice in PBS and permeabilized with NET-gel (5 mM EDTA, 50 mM Tris–HCl pH 7.4, 0.05% NP-40, 0.25% carragenin lambda gelatin; 0.02% NaN_3_) for 40 min. The short incubation time, quite comparable to that used for HUVEC cells in some studies (6 h) [10], best suited the rapid self-organization of PGL cells into a web-like network. Primary antibodies to CD34 (Ancell mouse monoclonal #183-040), CD31 (BD Pharmigen mouse monoclonal #555445), DLK1 (Abcam mouse monoclonal #119930) and platelet-derived growth factor receptor alpha (PDGFRA, Santa Cruz rabbit polyclonal sc-338) were diluted in NetGel (1:50 except anti-DLK1, 1:500) and incubated overnight. After washing twice with NetGel, goat anti-mouse (Invitrogen #A11017) or goat anti-rabbit (Invitrogen #A11070), both diluted 1:100 in NetGel, were incubated for 1 h, washed once with NetGel and once with PBS and examined with an LSM 510 confocal microscope (Zeiss). The neuroblastoma cell line SH-SY5Y (ATCC; CRL-2266), acquired in 2007 and authenticated in January 2013 [7], was cultured in RPMI 1640 (GE Healthcare) containing 10% FBS, 2 mM l-glutamine, 100 IU penicillin, 100 μg/ml streptomycin [7]. Control human embryo lung fibroblast MRC5 cells, obtained from Karolinska Institutet, Stockholm, were cultivated in eMEM containing 10% FBS and 2 mM l-glutamine, 100 IU penicillin, 100 μg/ml streptomycin.

### Ex vivo cultures and xenografts

For ex vivo culture PGL samples taken at surgery in high-glucose DMEM with penicillin, streptomycin and fungizone were cut into ~ 3 × 3 mm cubes under sterile conditions and cultured at 37 °C in 12-well plates (as used for cells) for up to 10 days. No enzymatic dissociation was used. Samples were then processed and analysed as described above. To obtain PDXs, fresh PGL tissues, sampled and cut as above, were transplanted subcutaneously within 24 h from surgery into the flank(s) and/or under the neck skinfold(s) of 6–12 weeks old NOD scid gamma (NSG) mice (when possible sex-matched to the donor patient). Normal tissues (abdominal skin) from PGL patients were transplanted as controls. The immortalized PTJ64i PGL cell line, that can be grown in large quantities, was used to obtain cell-derived xenografts (CDXs). To this end athymic female CD-1 nude mice aged 4 weeks were acquired from Charles River and, after 2 weeks of quarantine, grafted in both flanks with 9 × 10^6^ PTJ64i cells in 0.1 ml PBS. All mice were maintained in temperature-controlled cages docked on a ventilated rack with HEPA filters, euthanized by CO_2_ inhalation followed by exsanguination via cardiac puncture and, in some cases, intracardiac perfusion with 1 ml India ink solution (1/20 in sterile H_2_O with 10 IU/ml heparin), to evaluate the integration between PDX and murine vasculature. Mice were finally dissected to identify putative growths in transplant areas, that were measured, photographed and, depending on tissue availability, sampled as described for PGLs. The thoracoabdominal organs were routinely examined and sampled for standard microscopy.

### Mt DNA analysis

DNAs from PDXs, blood of the donor patients and host mouse tissues were extracted using the PureLink Genomic DNA Kit (Invitrogen) and quantified with Nanodrop 2000 (Thermo Scientific). HVR1 mtDNA typing was performed via PCR and direct automated sequencing using primers L15990 (sense) and H16434 (antisense) [56]. Sequences were aligned and compared to the HVR1 consensus reference sequence.

### JC-1 staining

The cell-permeant vital dye JC-1 (5,5′,6,6′-tetrachloro-1,1′,3,3′-tetraethylbenzimidazolylcarbocyanine iodide, Invitrogen) is concentrated in mitochondria with functional OXPHOS. This results in a fluorescence emission shift from green (~ 529 nm) to red (~ 590 nm), due to concentration-dependent formation of intramitochondrial J-aggregates that accurately reflect the mitochondrial membrane potential (mt ΔΨ) [52]. For JC-1 staining PGL tissues and autologous controls (subcutaneous white adipose tissue, WAT) were obtained at surgery, maintained in high-glucose DMEM with penicillin, streptomycin and fungizone for up to 24 h, minced aseptically in ~ 3 × 3 mm fragments, incubated for 10 min at 37 °C in culture medium containing 15% FBS and 5 µM JC-1, washed for 1 min with warm PBS and placed in Cell Imaging Dishes (Eppendorf). Then the red/green fluorescence intensity ratios, that are specific indicators of mt ΔΨ [52], were independently calculated for the vascular/glial and the neuroepithelial PGL components and compared to the red/green fluorescence ratios calculated for the WAT controls. PGL cell cultures and control MRC5 cells, live on CultureSlides (BD Falcon), were JC-1 stained in PBS plus 5 µM DRAQ5 (Thermo Fisher Scientific) to counterstain nuclei. Tissues and cells were analysed with an LSM 510 confocal microscope (Zeiss).

### Imatinib treatments

Stock solutions of imatinib (Selleck Chemicals) in sterile H_2_O (200 mM) were stored at − 20 °C. The imatinib concentration for in vitro treatment (10 μM) was chosen based on IC50 values determined on PGL cell cultures using the Alamar Blue cell viability assay (Invitrogen) and the trypan blue exclusion method [18]. Imatinib or an equal volume of sterile H_2_O were added to triplicate wells of 24-well plates seeded with 5000 cells. Cells were incubated in a humidified chamber at 37 °C-5% CO_2_ with imatinib- or H_2_O-supplemented medium replaced every 24 h and counted at 24, 48 and 72 h using the trypan blue exclusion method. Significance was determined by two-way ANOVA with 95% CI and Bonferroni correction, statistical analyses were performed with Prism 6 (GraphPad Software, San Diego, CA). Caspase activity (average of triplicates normalized to controls) was evaluated with the Caspase-Glo 3/7^®^ Assay (Promega) using a Veritas microplate luminometer (Turner BioSystems). Significance was calculated with the unpaired *t* test. Treated and control cultures were analysed by AIF and WB for ZEB1 and PDGFRA, by AIF for BAX, and by WB for antiphosphotyrosine, PDGFRB, Beclin 1, vinculin, and β-actin. Antibodies were as reported above. MicroRNA levels before and after treatment were evaluated as described [7].

In vivo prevention of CDX formation by imatinib was evaluated on 11 female CD-1 mice (Charles River) grafted in both flanks with 9 × 10^6^ PTJ64i cells in 0.1 ml PBS at 6 weeks of age. The mice were randomly assigned to two groups and injected i.p. once daily, starting day 3, with either a preselected bulk dose of imatinib in sterile H_2_O (50 mg/Kg, Biorbyt, Cambridge, UK), adapted from the literature [13] (5 mice, 10 grafts), or an equal volume of sterile H_2_O (6 mice, 12 grafts), for 20 days. Treatment was then scaled-down to a one-third dose (16.6 mg/Kg) for 20 additional days, after which the mice were euthanized, analysed and sampled as above. Body weight was measured weekly throughout treatment. Autopsy and standard microscopic analysis of the thoraco-abdominal organs were routinely performed.

## Results

### Characteristics of the tumours

Background information on the examined series of 77 head and neck PGL cases, including *SDHx* mutation carrier status of the patients and tumour immunostaining for the SDHB protein, surrogate marker of *SDHx* mutations [24], are described in the Online Resource 1 (Tables S1–S3). All the tumours were immunophenotyped using standard IHC on paraffin sections and AIF on semithin cryosections. The fine morphology was routinely investigated by TEM.

**Fig. 1:**
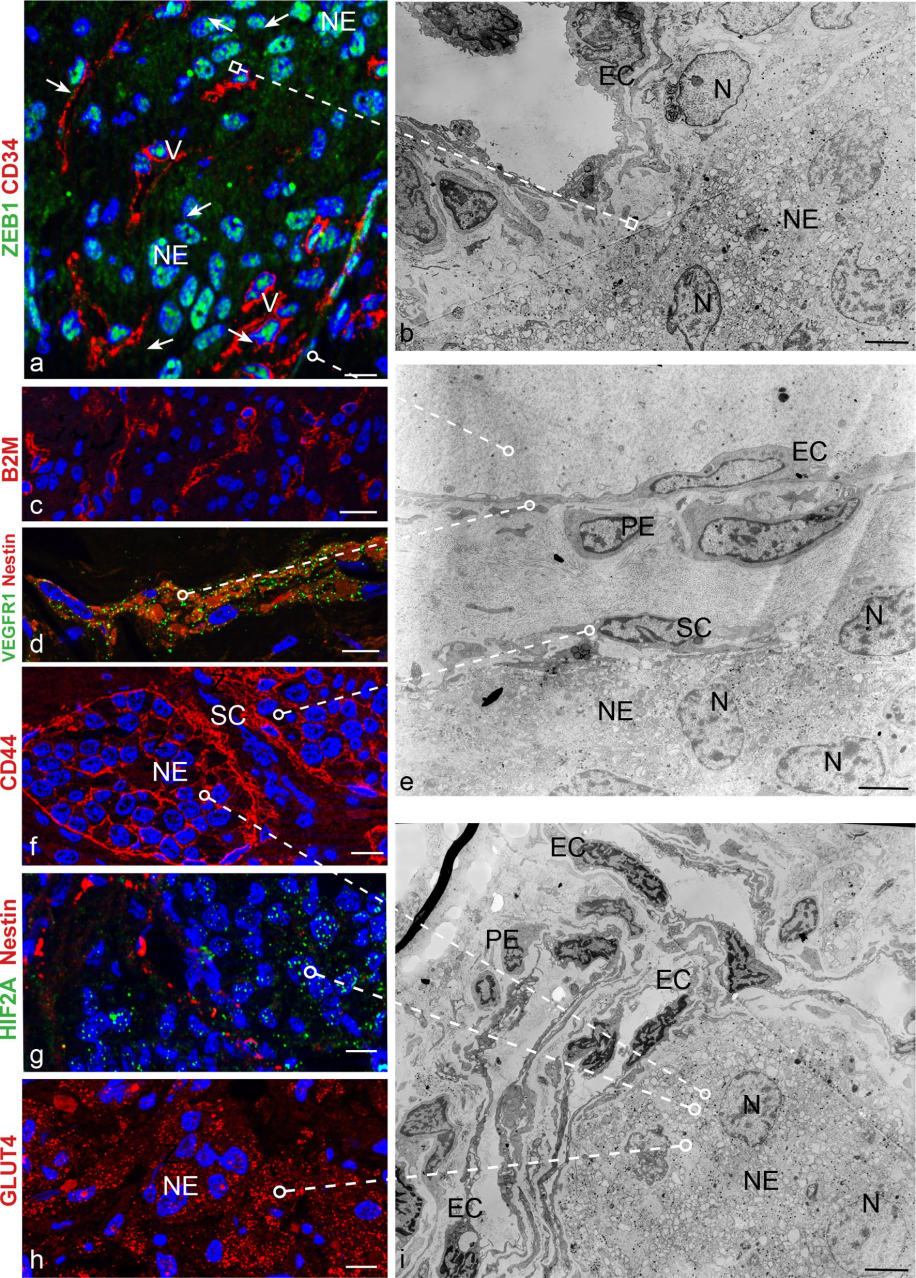
The vasculoneural paraganglioma network expresses mesenchymal, hypoxic, vasculoneurogenic and metabolic markers and is ultrastructurally aberrant. **a** ZEB1 is evident in the nuclei of both neuroepithelial (NE) and vascular cells (V), the latter identified by proximity to CD34-positive capillaries. **b** Ultrastructural relationship between an atypical capillary, with prominent endothelial cells (EC), and a cohesive nest of neuroepithelial cells (NE) with vacuolated cytoplasm, swollen mitochondria, neurosecretory granules and notched, lightly clumped nuclei (N). **c**–**d** Vascular distribution of β2-microglobulin (B2M), nestin and VEGFR1. **f** The hyaluronan receptor CD44 outlines the membranes of sustentacular (SC) and neuroepithelial (NE) zellballen cells. **e** Ultrastructural relationships between the four major cell types that organize the paraganglioma microenvironment: *EC* endothelial cells, *PE* pericytes, *SC* sustentacular cells, *NE* neuroepithelial cells. **g** Nuclear and cytoplasmic HIF2A is mainly evident in nestin-negative non-vascular cells. **h** Strong expression of the the insulin-regulated glucose transporter GLUT4 is mainly evident in neuroepithelial cells (NE). **i** Ultrastructural context showing neuroepithelial nests (NE) and adjacent capillaries with endothelial cells (EC) and pericytes (PE). DAPI (blue) identifies nuclei in the ApoTome immunofluorescence panels, dashed lines link immunofluorescence images to comparable ultrastructural areas. Bars = 10 µm (ApoTome immunofluorescence) and 5 µm (transmission electron microscopy). *N* nuclei

By IHC markers associated with endothelial (CD34), pericytic (SMA), glial (S100) and neuroepithelial (SYP) differentiation clearly discriminated the four major cell types that define the PGL microenvironment, but we observed that these otherwise phenotypically distinct cell types coexpressed to variable extents the mesenchymal markers vimentin and NOTCH1 (Online Resource 2, Fig. S1). AIF analyses confirmed that PGL cells of vascular and neural lineage always coexpressed immature markers associated with mesenchymal stem cells (CD44, vimentin) [14, 74, 75], the hypoxia-ZEB1 axis (HIF2A, ZEB1, NOTCH1, DLK1) [33, 37, 41, 42, 59, 70, 77], glucose uptake (GLUT4) [59] and vasculoneurogenesis (NOTCH1, DLK1, nestin, PDGFRA, KIT/CD117, VEGFR1, VEGFR2) [9, 11, 31, 38, 64] (Table 1, Fig. 1). Other markers were lineage-specific, including β2-microglobulin, CD34 and CD31 for endothelial cells, SMA for pericytes; S100 and GFAP for glial (i.e., sustentacular) cells; CGA and β3tub for neuroepithelial cells (Table 1, Fig. 1). Both the vascular and the neural PGL components were ultrastructurally aberrant (Fig. 1).

**Table 1:** ApoTome immunofluorescence phenotypes of the cell types found in paragangliomas, derived adherent and neurosphere cell cultures and xenografts (21 markers)

	Paragangliomas				Cultures		Xenografts			
	Endothelial	Perivascular	Glial	Neuroepithelial	Adherent	Neurosphere	Endothelial	Perivascular	Glia-like	Neuroepithelial
HIF2A	+	+	+	+	+	+	+	+	+	+
ZEB1	+	+	+	+	+	+	+	+	+	+
CD44	–	–	+	+	+	+	NA	NA	+	+
NOTCH1	+	+	+	+	+	+	+	+	+	+
Vimentin	+	+	+	+	+^a^	+^a^	+	+	+	+
DLK1	+	+	+	+	+	+	–	+	+	+
PDGFRA	+	+	+	+	+	+	+	+	+	+
KIT	+	+	+	–	NA	NA	+	+	+	+
VEGFR1	+	+	+	+	+	NA	+	+	+	+
VEGFR2	+	+	+	+	+	+	+	+	+	+
Nestin	+	+	+	–	+^a^	+^a^	+	+	+	–
GLUT4	+	+	+	+	+^b^	+^b^	+	+	+	+
SMA	–	+	–	–	+	+	–	+	+	–
β2M	+	–	–	–	NA	NA	+	–	–	–
CD34	+	–	–	–	±^c^	±^c^	+	–	–	–
CD31	+	–	–	–	NA	NA	+	–	–	–
GFAP	–	–	+	–	+	NA	–	–	+	–
S100	–	–	+	–	NA	NA	–	–	+	–
β3Tub	–	–	+	+	+	+	–	–	+	+
NCAM	–	–	+	+	±^c^	NA	–	–	+	+
CGA	–	–	–	+	–	NA	–	–	–	–

*NA* not assessed

^a^Vimentin positive cells show low or no Nestin, and viceversa

^b^Prevalently nuclear staining

^c^Membrane and cytoplasmic staining in few cells only

Ex vivo flow cytometry further substantiated the presence of consistent PGL cell populations positive for mesenchymal and stem cell markers (CD44, CD73, CD90, CD105, CD133) [14, 74, 75] (Online Resource 1, Tables S4, S5; Online Resource 2, Fig. S2) and demonstrated that variable subsets of PGL cells expressed hematopoietic/endothelial (CD34), glial (GFAP) and/or neural (NCAM) stem/progenitor cell markers. However, in most of the examined cases, the cells positive for CD34 comprised subsets coexpressing stem cell (CD133/CD44), neural (NCAM) or glial (GFAP) markers, suggesting phenotypic transition potential (Online Resource 1, Tables S5, S6).

These findings, replicated in tumours from *SDHx* carriers and noncarriers (Table 1; Online Resource 1, Tables S1, S5, S6), indicate that PGLs, regardless of the *SDHx* genetic background of the patient, contain immature cells with hybrid mesenchymal–vasculoneural features, likely primed for vascular (endothelial/mural) or neural (glial/neuroepithelial) differentiation.

**Fig. 2:**
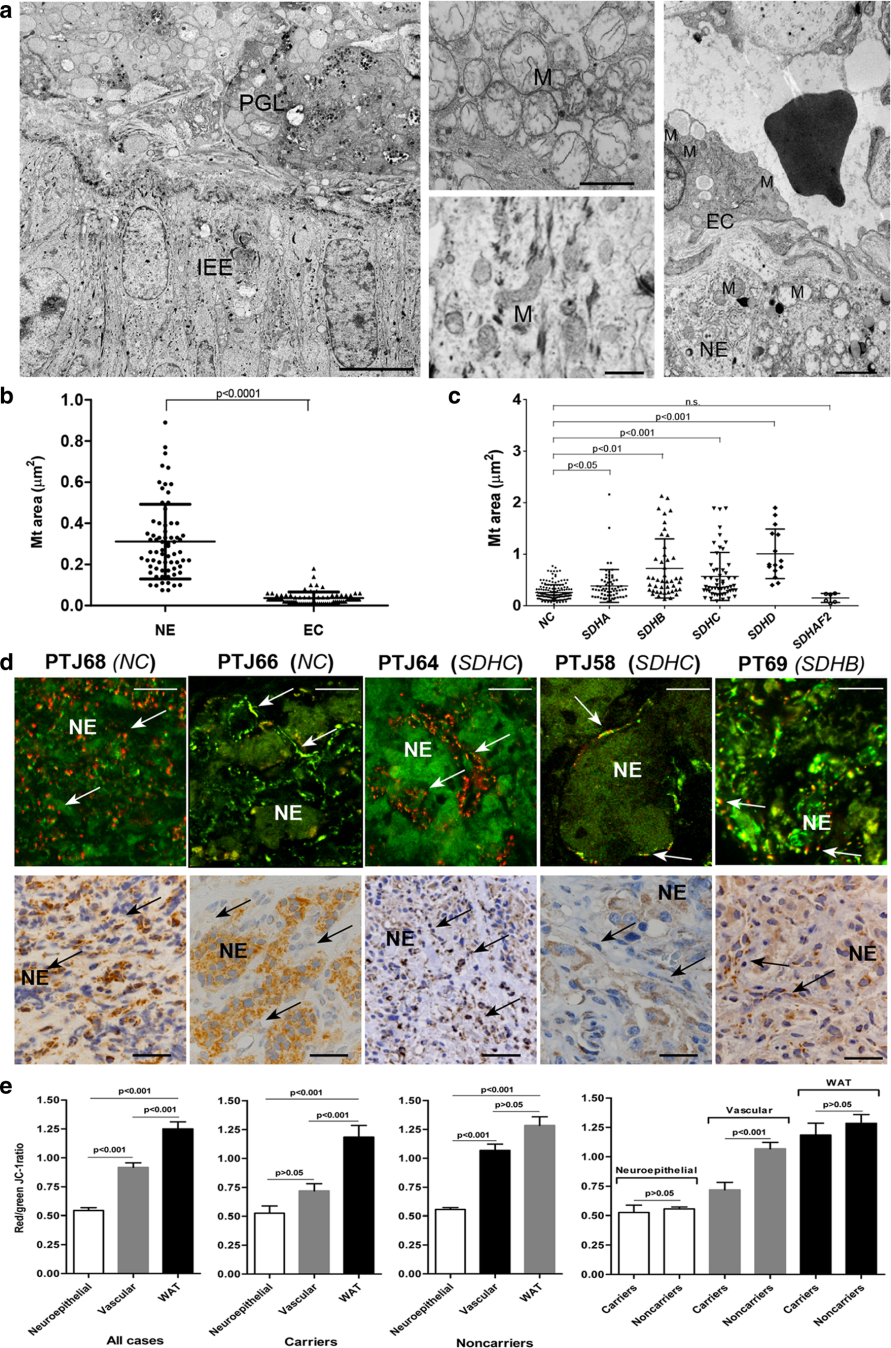
Morphofunctional alterations of mitochondria in the neuroepithelial component of paraganglioma. **a** The transmission electron micrograph on the left shows the interface between normal inner ear epithelium (IEE, bottom half of the microscopic field) and an underlying non-embolized tympanic area of a tympano-jugular paraganglioma (PTJ64, from an *SDHC* mutation carrier, top half). The central higher magnification views illustrate the ultrastructure of the mitochondria in IEE cells (bottom) and in neuroepithelial paraganglioma cells (top). Mitochondrial hyperplasia with swelling and consequent loss of cristae is clearly evident in paraganglioma cells, while the mitochondria of the adjacent inner ear epithelial cells are structurally normal, which rules out the possibility that the mitochondrial alterations might have been due to surgical/post-surgical manipulation. The transmission electron micrograph on the right, focused on another PTJ64 paraganglioma field, illustrates the morphological differences between the roundish, swollen mitochondria with disrupted cristae routinely found in neuroepithelial cells (NE) and the smaller, relatively well-preserved mitochondria found in the adjacent endothelial cells (EC). The capillary lumen contains an erythrocyte. Bars = 10 µm (left image) and 1 µm (centre and right images). **b** Scatter plots of mitochondrial cross-sectional areas (µm^2^) determined on electron micrographs of neuroepithelial (NE) and endothelial cells (EC) from 20 paragangliomas (69 measurements randomly extracted from a total of 354 measurements available for NE cells compared to 69 measurements for endothelial cells). **c** Scatter plots of mitochondrial cross-sectional areas (µm^2^) determined on electron micrographs of neuroepithelial (NE) paraganglioma cells presented according to germline *SDHx* status. Mean areas were: 0.258 ± 0.150 µm^2^ (noncarriers, NC, 168 measurements); 0.384 ± 0.318 µm^2^ (mutated in *SDHA*, 66 measurements, *P* < 0.05); 0.724 ± 0.573 µm^2^ (mutated in *SDHB*, 45 measurements, *P* < 0.01); 0.568 ± 0.465 µm^2^ (mutated in *SDHC*, 54 measurements, *P* < 0.001); 1.008 ± 0.480 µm^2^ (mutated in *SDHD*, 15 measurements, *P* < 0.001); 0.152 ± 0.087 µm^2^ (mutated in *SDHAF2*, 6 measurements, *P* > 0.05). *P*-values were calculated using one-way anova with Games-Howell post hoc test. **d** JC-1 fluorescence highlights collapse of the mitochondrial membrane potential in the neuroepithelial component. A set of 5 paragangliomas is shown, two from *SDHx* noncarriers (PTJ68, PTJ66) and three positive for germline mutation in *SDHC* (PTJ64 and PTJ58) and *SDHB* (PT69). Regions of high mt polarization (top panels, red to yellow fluorescence, indicative of concentration-dependent J-aggregates, arrows) largely correspond to the vascular/glial component, while the neuroepithelial nests and cords (NE) are depolarized, as indicated by the green fluorescence due to JC-1 monomers. Micrographs in the panels below show SDHB immunohistochemistry for the same tumors, where arrows point to the vascular/perivascular component that supports more or less defined neuroepithelial zellballens (ZB). Brown granular SDHB staining is undetectable in the neuroepithelial components of PT69, whereas PTJ58, PTJ64, PTJ66, PTJ68 are weakly to strongly positive. The variability in SDHB protein immunohistochemistry contrasts with the constant evidence of mt depolarization in the neuroepithelial component, which is unrelated to *SDHx*/SDHB status. Bars = 20 µm. Hematoxylin is used as a counterstain in SDHB immunohistochemistry. **e** The red/green JC-1 fluorescence ratios, indicative of mt membrane potential, decrease in the vascular (mean: 0.92 ± 0.04, all cases; 0.72 ± 0.06, carriers; 1.07 ± 0.05, noncarriers) and, more prominently, neuroepithelial (mean: 0.54 ± 0.02, all cases; 0.53 ± 0.06, carriers; 0.56 ± 0.02, noncarriers) paraganglioma components compared to the autologous white abdominal adipose tissue (WAT, mean: 1.25 ± 0.06, all cases; 1.18 ± 0.1, carriers; 1.28 ± 0.07, noncarriers). The differences between the neuroepithelial and the vascular tumour components and between the neuroepithelial tumour component and WAT are significant. Data are from 11 PGLs, of which 4 associated with identified germline *SDHx* mutations and 7 *SDHx* mutation-negative

It has long been known that PGLs may harbour ultrastructural mitochondrial aberrations predictive of reduced OXPHOS [69]. We, therefore, investigated the fine mitochondrial ultrastructure of the various PGL cell types. This revealed that hyperplastic and swollen mitochondria with disrupted cristae selectively accumulated in the neuroepithelial cells, a characteristic found in all the 77 PGLs examined by TEM (Fig. 2a, b). Comparing the cross-sectional areas of the mitochondrial profiles, it was nonetheless evident that larger mitochondria were significantly associated with PGLs from carriers of mutations in *SDHB*/*C*/*D* and to, to a lesser extent, *SDHA*, while the mitochondria associated with *SDHAF2* mutation carrier status (unique case) were actually smaller than those of the PGLs from noncarriers (Fig. 2c). We next assessed the mt ΔΨ of the PGL tissue components using the JC-1 assay. The red-to-green fluorescence ratios, which reflect the mt ΔΨ [52, 73], decreased in the vascular PGL component relative to the autologous adipose tissue control and collapsed in the neuroepithelial component (Fig. 2d, e). This occurred in all of the 11 PGLs tested, with non-significant differences between the seven tumors from *SDHx* carriers and the four tumors from non-carriers (Fig. 2e). Thus, the neuroepithelial PGL lineage was characterized by severe mitochondrial dysfunction, implying that OXPHOS inactivation depended on the differentiation and/or the microenvironment.

### Patient-derived paraganglioma cultures

**Fig. 3:**
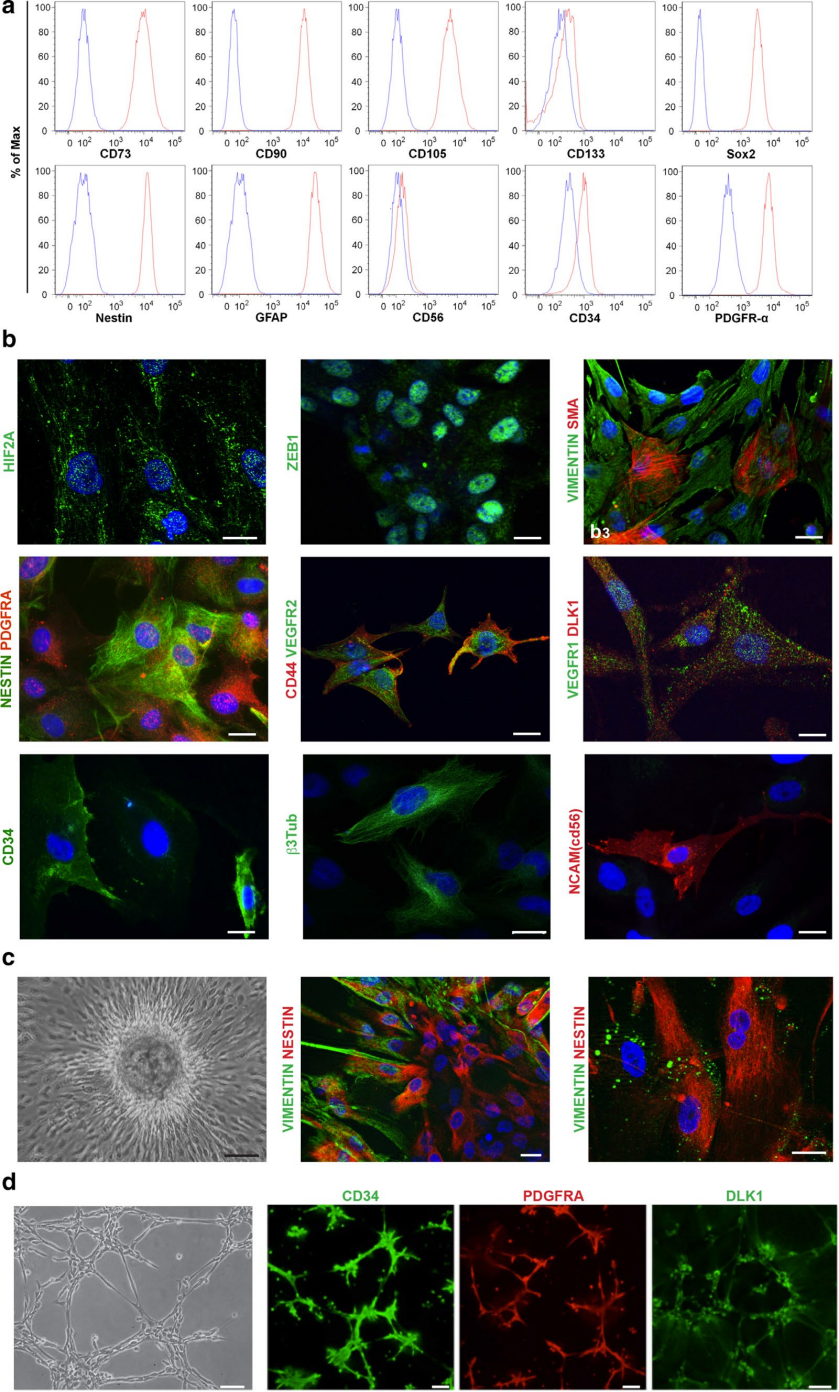
Characteristics of the paraganglioma cell cultures. **a** Flow cytometry phenotype based on the expression of surface related (CD73, CD90, CD105, CD133, NCAM/CD56, CD34, PDGFRA) and intracellular (SOX2, Nestin, GFAP) antigens in a primary paraganglioma cell culture (PTJ114p, passage 6). Red and blue histograms show the distribution of antigen expression and background control, respectively. Data are representative of experiments performed on 18 primary and four immortalized paraganglioma cultures (see Online Resource 1, Table S7). **b** ApoTome immunofluorescence analysis of representative paraganglioma cell cultures PTJ64p and PTJ86p illustrates positivity for: HIF2A (cytoplasmic and nuclear); ZEB1 (nuclear); smooth muscle actin (SMA) versus vimentin; nestin (filamentous cytoplasmic) versus PDGFRA (punctate pattern: membrane, cytoplasm, nucleus); VEGFR2 (membrane, cytoplasm and nucleus) versus CD44 (membrane and cytoplasmic); VEGFR1 versus DLK1 (both with membrane, cytoplasmic and nuclear distribution); CD34 (membrane and cytoplasmic); β3-tubulin (filamentous cytoplasmic); NCAM/CD56 (membrane and cytoplasmic). DAPI is used as a nuclear marker, bars = 10 µm. **c** Tridimensional growth in a primary culture (PTJ64p). From left to right: tridimensional focus at subconfluence (phase contrast); optical ApoTome immunofluorescence section illustrating vimentin positivity in fusiform peripheral cells and nestin positivity in inner cells; higher magnification field showing residual vimentin in cytoplasmic vacuoles of nestin-positive cells, suggestive of lysosomal degradation. DAPI is used as a nuclear marker, bars = 30 µm (left) and 10 µm (center and right). **d** Vasculoangiogenic potential. From left to right: phase contrast microscopy shows that already at 4 h from seeding on Matrigel paraganglioma cultures self-organize into a web-like network suggestive of microvascular capillary cords; confocal microscopy illustrates positivity for CD34, PDGFRA and DLK1. Images are from PTJ84p (from an *SDHx* noncarrier), similar results (not shown) were obtained for PTJ62p, PTJ66p, PTJ67p (all from noncarriers) and PTJ86p (from an *SDHD* mutation carrier). Bars = 100 µm

We developed 18 primary and 4 immortalized cell cultures from 19 PGLs (Online Resource 1, Table S7). Compared to the dissociated PGL cells, these cultures demonstrated quite homogeneous flow cytometric profiles, characterized by diffuse and mostly strong positivity for the mesenchymal markers CD73, CD90, CD105, for the embryonic and neural stem cell markers SOX2 and nestin, and for GFAP and PDGFRA. CD34 and NCAM were less consistently expressed; CD133 was below detection level (Fig. 3a; Online Resource 1, Table S7). These characteristics were unaffected by immortalization and by the *SDHx* carrier status of the donor patient (Online Resource 1, Table S7).

By AIF the PGL cultures, both primary and immortalized, diffusely exhibited the immature mesenchymal, hypoxic and vasculoneural markers shared by the vascular and neural components of the PGLs of origin, CD34 was detectable in few cells, β3tub was heterogeneously expressed, CGA was undetectable (Fig. 3b; Table 1). In the tridimensional foci randomly formed in adhesion vimentin was expressed in cells of the outer shell, SMA and nestin in inner cells, not directly exposed to the culture medium (Fig. 3c). When plated on Matrigel [19] the cells rapidly generated pseudovascular networks expressing the putative endothelial marker CD34 together with DLK1 and PDGFRA, key components of the signalling mechanisms that engage mural cells to nascent endothelial tubes [11, 37, 55, 62, 64] (Fig. 3d). Under non-adhesive conditions the cultures formed neurospheres (Online Resource 2, Fig. S3a), that strongly expressed the stem/mesenchymal markers found in the original PGL biospecimens and in the adherent cultures (Table 1; Online Resource 2, Fig. S3b). Notably the cultured cells, both primary and immortalized, contained ultrastructurally normal tubular mitochondria with mainly red JC-1 fluorescence (high mt ΔΨ), implying functional OXPHOS (Online Resource 2, Fig. S4). Thus, the mitochondrial dysfunction noted in the neuroepithelial component of the PGLs was not conserved in the mesenchymal-like cells that developed under culture conditions.

### High ZEB1 and PDGFRA levels in PGL cells are a consequence of microRNA dysregulation

**Fig. 4:**
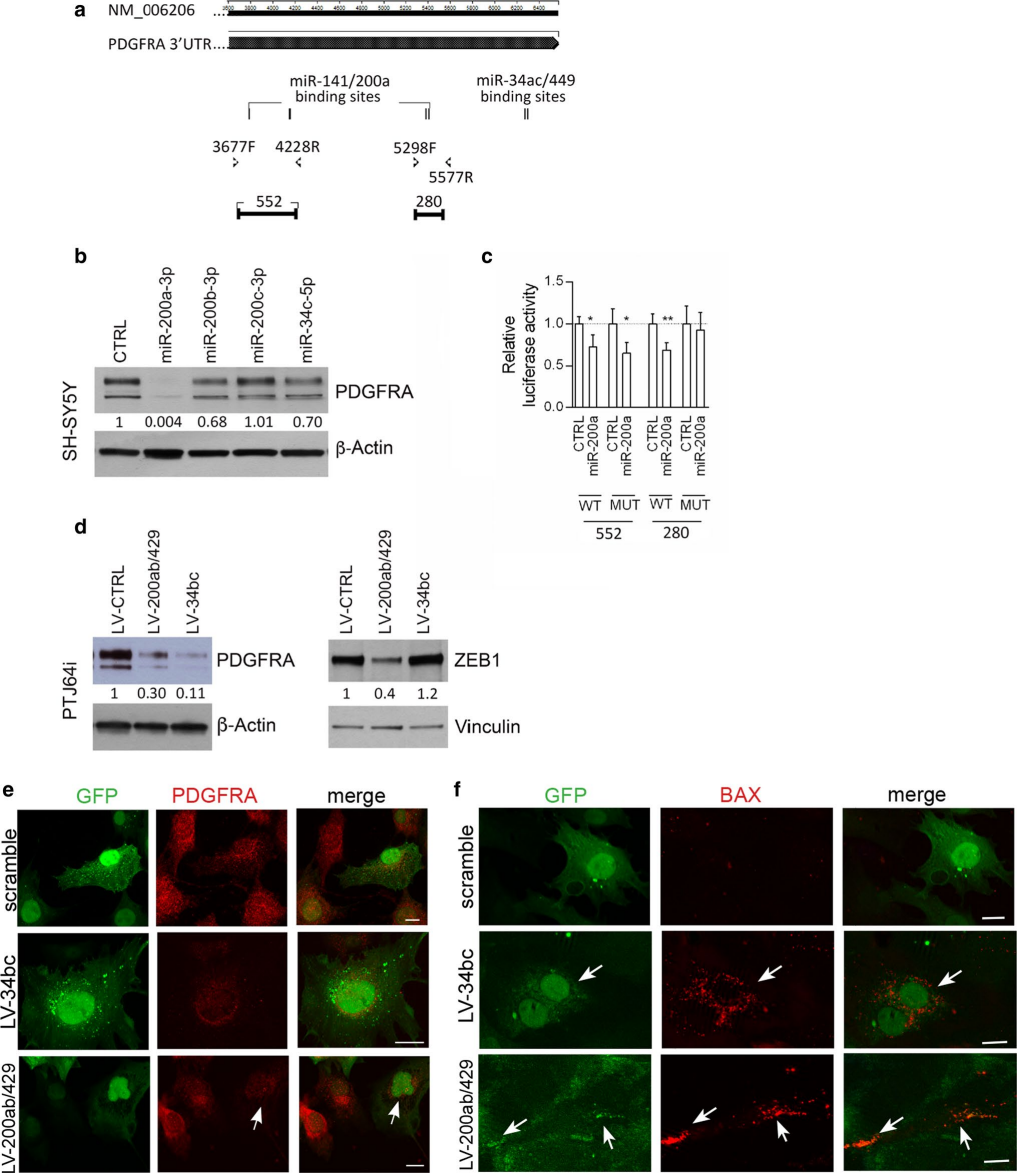
MiR-200 and miR-34 family members target PDGFRA and ZEB1 in paraganglioma cells and induce cell death. **a** Schematic structure of the PDGFRA cDNA with location of four putative binding sites for miR-141/200a and of two for miR-34ac/449 (Targetscan). **b** Western blot analysis of PDGFRA (upper panel) and β-actin (loading control, bottom panel) after the indicated microRNA mimic transfections in SH-SY5Y neuroblastoma cells. Normalization with densitometric analysis is shown. **c** Relative luciferase activity dependent on miR-200a mimic transfections in SH-SY5Y cells is also shown (*WT* wild type, *MUT* mutant). **d** Western blot analysis of PDGFRA, ZEB1 and loading controls (β-actin and vinculin) in PTJ64i cells infected with lentivirus transducing either miR-200s or miR-34s or scramble. Normalization with densitometric analysis is shown. **e**, **f** ApoTome immunofluorescence, which distinguishes lentivirus-infected (GFP-positive) from non-infected (GFP-negative) PTJ64i cells, shows loss of PDGFRA and BAX expression in GFP-positive cells infected with lentivirus transducing miR-34bc or miR-200ab/429 compared to scramble control

Our previously reported evidence that the miR-200a,b,c and the miR-34b are consistently downregulated in PGLs [7] contributes to explain why PGLs and derived cell cultures express constitutively high levels of two proteins that are known to be targeted by these miRs, NOTCH1 and ZEB1 [7, 70]. We previously validated the *NOTCH1* gene as a miR-200 and miR-34 target in PGL cells [7], but *ZEB1* remained to be confirmed in the specific context. Considering additional targets, we noted that the *PDGFRA 3′UTR* (cDNA NM-006206) contains four predicted binding sites for the miR-141/200a (Fig. 4a) and that miR-34a and -34c reportedly downregulate PDGFRA in lung cancer cells [21]. Using SH-SY5Y neuroblastoma cells, transfectable with miR mimics, we demonstrated that PDGFRA protein expression decreased by about 30% with miR-34c-5p mimic and by 99.96 and 32% with miR-200a-3p and miR-200b-3p mimics, respectively. No reduction was observed with miR-200c-3p mimics (Fig. 4b). To test direct interactions with the *PDGFRA 3′UTR*, the predicted wild-type and mutant miR-200a-3p target sites of the *3′UTR*-*PDGFRA* cDNA were cloned downstream of the psiCHECK2 luciferase reporter vector and co-transfected with miRNA mimics into SH-SY5Y cells. Significant reductions in the luciferase activity of the vector carrying the wild-type *PDGFRA*-*3′UTR*, confirmed at three of four sites (Fig. 4c), demonstrated that miR-200a-3p directly targets *PDGFRA*.

To validate *ZEB1* and *PDGFRA* as miR-200 and/or miR-34 targets in a PGL cell context, we enforced miR-200 (LV-200ab/429) or miR-34 (LV-miR-34bc) overexpression by lentiviral infection in PTJ64i cells. As expected, miR-200ab/429 reduced both the PDGFRA and the ZEB1 protein levels (70 and 60% respectively), while miR-34bc strongly reduced the PDGFRA protein level (89%), but did not affect ZEB1 (Fig. 4d). AIF analysis corroborated these data, showing that the loss of PDGFRA signal following the reintroduction of the miRs was accompanied by an upregulation of BAX protein expression, which pointed to the activation of an apoptotic response (Fig. 4e, f).

### PDXs arise via vasculo-angio-neurogenesis from mesenchymal-like cells

PDX formation is predicted to depend on cells that elude ischemic necrosis, a consequence of prolonged devascularization during heterotransplantation and engraftment [26, 44, 63]. To highlight such cells, we analysed ex vivo cultured PGL tissues, a model that mimics the microenvironment of the post-transplantation phase preceding xenograft vascularization. Confocal and semithin section light microscopy coupled with TEM showed that by day 10 post-surgery the tumour tissue underwent widespread coagulative necrosis with loss of all vascular and neural cells, but some areas were recolonized by vimentin-positive mesenchymal-like cells, characterized by pseudopodia and filopodia, indicative of motility and phagocytic capability (Online Resource 2, Fig. S5a). Such endogenous cells remodelled the necrotic scaffold through phagocytosis and deposition of collagenous matrix and formed CD34-positive pseudovascular structures. These were the only cells identifiable in the ex vivo-cultured PGL samples and were similar to those observed in an early phase of PDX development (3 weeks; Online Resource 2, Fig. S5b), which suggests that they correspond to the cells responsible for PDX initiation.

We next developed PDX models to investigate PGL development in vivo. A total of 90 PGL fragments from 16 patients were xenografted into immunodeficient mice, with a high overall take-rate of 89% (80/90), not affected by the *SDHx* mutation carrier status of the donor patient (Online Resource 1, Table S8).

At harvest, 4.5–10 months post-transplantation, the PDXs presented as ~ 4–6 mm nodules, which infiltrated the adjacent murine neurovascular bundles, as typical of PGLs [47, 53, 61] (Online Resource 2, Fig. S6a). The relatively small size was consistent with the well-known slow growth of PGLs in patients (0–2 mm/year) [47, 53, 61]. The PDX tissue, including the vasculature, was of human origin, as proved with human-specific antibodies and mt DNA analysis, and was linked to the systemic murine circulation, as demonstrated by its permeation with India ink solution after intracardiac perfusion (Online Resource 2, Fig. S6).

**Fig. 5:**
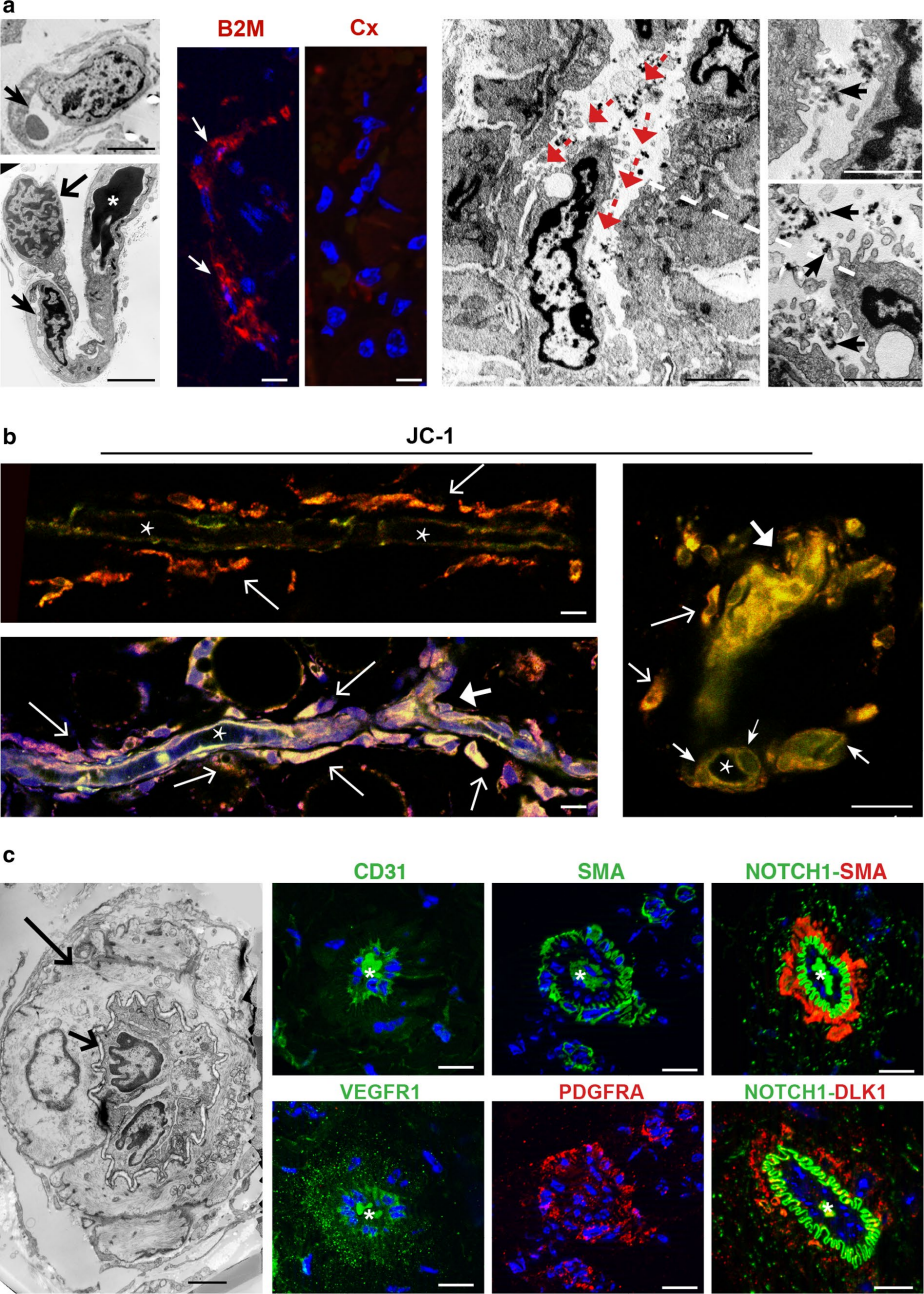
Patient-derived xenografts reveal endothelial tubulogenesis through cytoplasmic hollowing and vascular ramification via intussusceptive angiogenesis. **a** The transmission electron micrographs on the left show an early stage of lumenization, where endothelial precursors, with expanding cytoplasmic hollows (narrow arrows), associate (broad arrow) and coalesce in tubes (asterisk marks electron-dense, irrelevant intralumenal proteinaceous material). The semithin frozen section ApoTome images on the centre illustrate β2-microglobulin (B2M) positivity in a de novo formed endothelial tube versus control serial section (Cx). The transmission electron micrographs on the right illustrate intussusceptive pillar formation due to an endothelial infolding that splits the lumen into two newly formed lumens (red arrows indicate divided flow). Intraluminal India ink particles, visible in the higher magnification views on the far right (black arrows), indicate that the vessel undergoing remodelling is connected to the systemic murine vasculature. A dashed line links the tip of the intussuscepted endothelial cell to its context. Bars = 10 µm. **b** Mural cell recruitment and dichotomic vascular branching. Intravital whole mount JC-1 confocal microscopy illustrates lumenized endothelial tubes (asterisk: longitudinal or cross sections of vascular lumens) that attract assumably motile perivascular mesenchymal-like cells. Such cells, that have functional mitochondria, as indicated by JC-1 staining, are caught in the process of adhesion to the ablumenal side of the endothelial tubes (thin arrows). Recruitment of perivascular cells is associated with Y-shaped vascular branching (thick arrows). Bars = 10 µm (left images) and 20 µm (right image). **c** Characterization of the interaction between endothelial and mural cells during xenograft vasculogenesis. The transmission electron microscopy image on the right illustrates a vascular cross-section, with prominent mural cells, characterized by ovoid, scarcely clumped nuclei and clear, microfilament-rich cytoplasm (long arrow). Such cells wrap two smaller, darker endothelial cells that delimit a contracted lumen (short arrow). The pericyte-endothelial contact follows a jagged line (short arrow), where the endothelial and the mural plasma membranes are separated by a narrow gap. By ApoTome immunofluorescence, serial semithin sections of a similar vascular cross section are labelled with: CD31, which stains the endothelial cells only; smooth muscle actin (SMA), which stains the mural cells; NOTCH1 and SMA, which highlight the jagged abluminal endothelial membranes and the mural cells, respectively; VEGFR1, which stains both the mural cells and the endothelium; PDGFRA, which stains the mural cells and some adjacent interstitial cells; and, finally, DLK1 and NOTCH1. DLK1, a noncanonical NOTCH ligand, labels the mural cells in contact with the NOTCH1-positive ablumenal endothelial membranes. Homogeneous staining of intralumenal proteinaceous amorphous material (asterisk) is a fluorochrome-related artefact. DAPI is used as a nuclear marker in the ApoTome immunofluorescence images. Bars = 5 µm (transmission electron microscopy) and 10 µm (ApoTome immunofluorescence)

TEM and AIF revealed the fine morphological details of the vasculo-angiogenic processes taking place in the PDXs (Fig. 5). These initiated in individual endothelial precursors, that progressively developed intracytoplasmic lumina through vacuolization (i.e., cell hollowing), as reported in HUVECs and in drosophila and zebrafish embryos [16, 32, 35, 65, 72], mutually interacted and coalesced, forming lumenized tubules positive for β2-microglobulin, CD31 and CD34, as PGL endothelium (Fig. 5a; Table 1; Online Resource 2, Fig. S6c). The self-assembly of these tubular endothelial structures defined a novel perivascular niche, readily populated by as yet undifferentiated mesenchymal-like cells that tended to adhere to the abluminal endothelial membranes. Whole mount confocal microscopy revealed that the adhesion process was associated with dichotomic tubular branching, a remodelling pattern consistent with intussusceptive angiogenesis, a major form of nonsprouting angiogenesis that allows rapid bifurcation of neoformed vessels via endothelial invagination [45] (Fig. 5b). Endothelial intussusceptions dividing the original lumen into two newly formed minor lumens were confirmed by TEM (Fig. 5a, right panels). Of note, plasma membrane NOTCH1 was uniquely associated with the ablumenal aspect of the endothelium, in direct contact with closely adherent perivascular cells that expressed the HIF-induced non-canonical NOTCH antagonist DLK1, a cancer pericyte-related antigen, together with SMA and PDGFRA (Fig. 5c) [6, 11, 27, 39, 55, 62]. Such cells revealed ultrastructural features of mural cells (vascular smooth muscle cells or pericytes) (Fig. 5c).

**Fig. 6:**
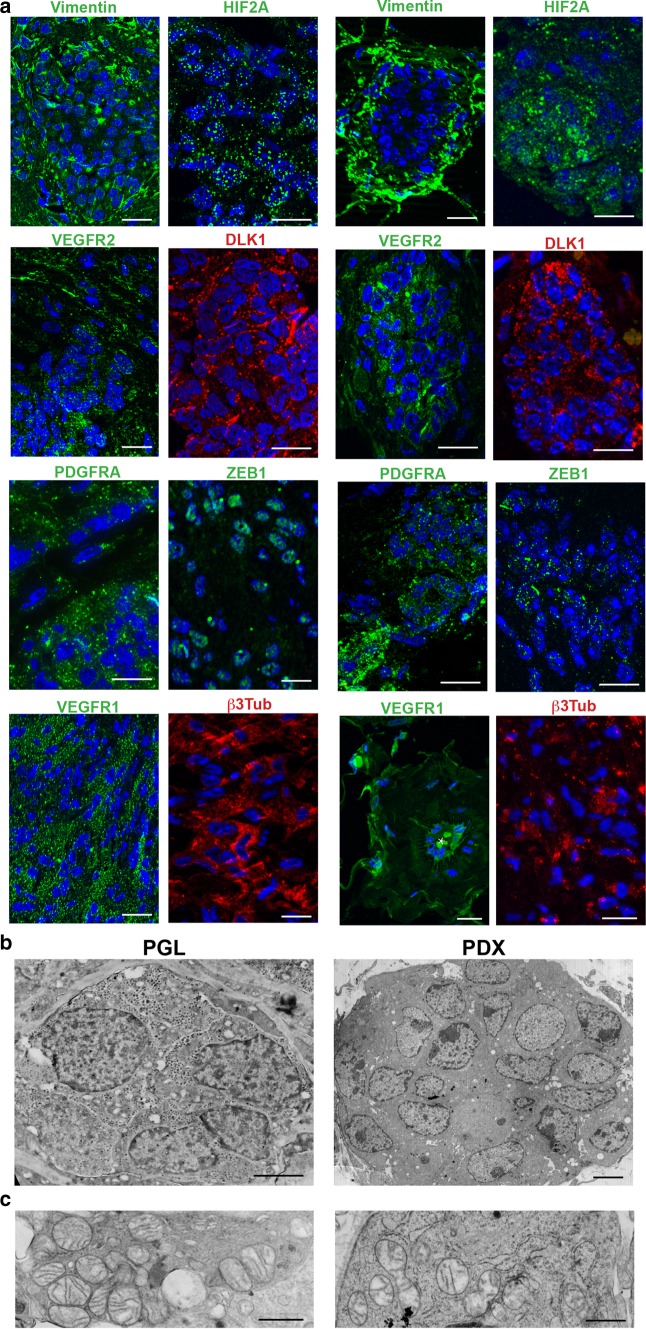
Ultrastructural and immunomorphological analogies between parangaglioma and derived xenograft. **a** ApoTome immunofluorescence of serial sections shows similar expression of vimentin, HIF2A, VEGFR2, DLK1, PDGFRA, ZEB1, VEGFR1 and β3-tubulin in paraganglioma (PGL) and patient-derived xenograft (PDX). Bars = 10 μm. DAPI is used as a nuclear marker. **b** Ultrastructural analogy between the neuroepithelial cell nest of a paraganglioma (PGL) and that of a corresponding patient-derived xenograft (PDX). The cohesive nests are structurally similar, except for the absence of neurosecretory granules in the PDX cells (Bars = 2 μm). **c** Similar mitochondrial alterations (swelling and loss of cristae) are visible in the neuropithelial cells of the paraganglioma (PGL) and of the derived xenograft (PDX) (Bars = 1.5 μm)

The early vasculo-angiogenic network developed on autonomously synthesized extracellular matrix (ECM) that initially contained collagen I, an EMT-related adhesive collagen associated with invasive growth and neovascularization [3, 51]. Then, with the recruitment of mural cells, collagen IV, main component of basement membranes [49, 60, 76], was deposited at the endothelial–mural interface and in the perivascular region (Online Resource 2, Fig. S7). This microenvironmental change was connected with the formation of clusters or ribbons of polygonal cells bound by glia-like spindle cells, that derived from the interstitial cells not involved in vascular differentiation. The de novo-formed cell nests were strongly reminiscent of the neuropithelial PGL component and exhibited the same immature mesenchymal, hypoxic and vasculoneurogenic markers found in neuropithelial PGL cells, together with β3Tub, despite negativity for the advanced neuroendocrine markers CGA and SYP (Fig. 6a, Table 1). Notably, the neuroepithelial-like cells of the PDX harboured hyperplastic and swollen mitochondria with disrupted cristae, indicative of context-dependent mitochondrial dysfunction, as in the neuroepithelial clusters of the PGLs (Fig. 6b, c).

### Imatinib blocks PGL cell growth and inhibits xenograft formation

**Fig. 7:**
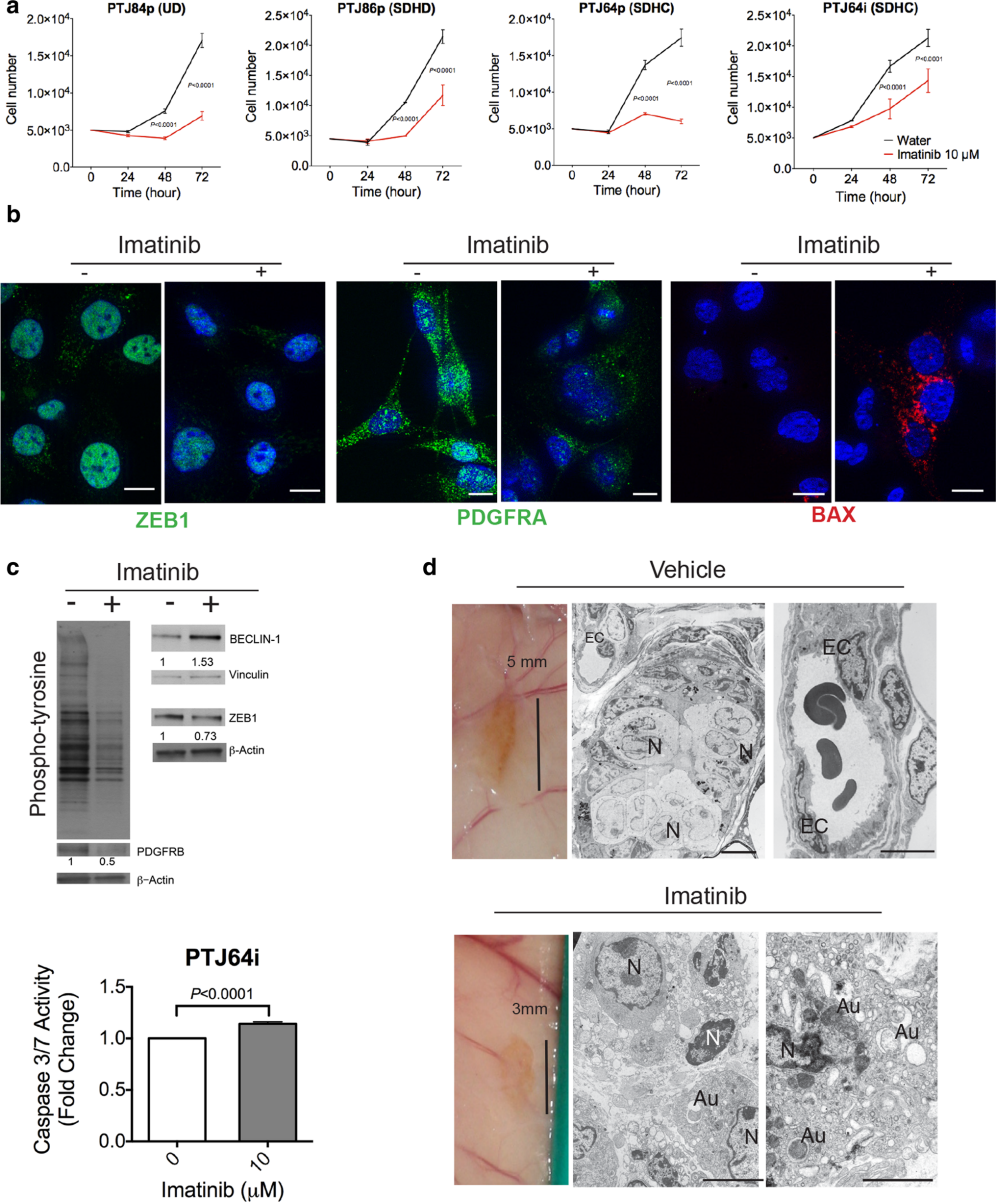
Imatinib arrests paraganglioma cell growth and prevents paraganglioma xenograft formation. **a** Imatinib at 10 μM concentration arrests the growth of paraganglioma cultures. Cultures include PTJ84p, from an *SDHx* noncarrier (NC); PTJ86p, mutated in *SDHD*; PTJ64p, mutated in *SDHC*, and PTJ64i, immortalized with SV40-hTERT. Results are means ± standard deviations of 3 replicates. **b** ApoTome immunofluorescence analysis. At 48 h of treatment with 10 μM imatinib versus vehicle PTJ64i cells show loss of ZEB1 and PDGFRA, while BAX becomes evident (DAPI is used as a nuclear marker, bars = 10 µm). **c** Western blot and caspase activity. Imatinib (10 μM) inhibits global protein tyrosine phosphorylation, downregulates ZEB1 and upregulates Beclin-1 in PTJ64i cells at 48 h of treatment (normalization relative to β-actin calculated with NIH ImageJ). In the same cells caspase 3/7 activity increases at 15 h. **d** Morphology of the PTJ64i cell xenografts formed in immunodeficient mice treated with imatinib versus vehicle. Tumour xenografts were identified at 2/10 PTJ64i transplant sites in the imatinib-treated group (50 mg/Kg for 20 days, then 16.6 mg/Kg for 20 additional days) versus 11/12 PTJ64i transplant sites in the vehicle-trated group (*P* = 0.0015). In the vehicle-treated group (upper panel images), the xenografts presented as brownish plaques within 5 mm of diameter. Transmission electron microscopy highlighted morphological features similar to those of the patient-derived tumour xenografts, with blood vessels lined by prominent endothelial cells (EC) associated with zellballen-like neuroepithelial nests delimited by glia-like fusiform cells. The far right image shows a well-formed blood vessel with endothelial (EC) and mural (MC) cells (*N* nuclei). The only two xenografts detected in the imatinib-treated group (exemplified in the lower panel images) were smaller in size (within 3 mm) and, by transmission electron microscopy, contained only poorly organized cells with severe cytoplasmic vacuolization and accumulation of autophagosomes (Au), suggesting suppressed autophagic turnover (*N* nuclei). There was no evidence of vascular and neuroepithelial structures

A low dose of imatinib (10 μM) significantly inhibited the growth of 4 PGL cell cultures (PTJ84p, PTJ86p, PTJ64p and the immortalized derivative PTJ64i), selected to represent PGLs from patients with diverse *SDHx* carrier status (Fig. 7a; cultures listed in Online Resource 1, Table S7). In PTJ64i cells, that could be grown in large numbers, downregulation of the ZEB1, PDGFRA and PDGFRB proteins, global protein dephosphorylation and an upregulation of Beclin 1 were evident at 48 h using ApoTome immunofluorescence and western blot (Fig. 7b, c). Activation of apoptosis was supported by evidence of BAX protein expression detected via ApoTome immunofluorescence at 48 h of imatinib treatment (Fig. 7b) and by a caspase assay at 15 h (Fig. 7c). Imatinib also induced significant mainly upward variation in the expression of miR-200a/b/c and miR-34b/c (Online Resource 2, Fig. S8), that may contribute to explain the downregulation of the ZEB1 and PDGFRA proteins.

We then verified whether early imatinib treatment, starting at day 3 from heterotransplantation, could inhibit in vivo xenograft formation. To this end PTJ64i cells were injected into the flanks of CD1 mice (11 mice, 22 transplants), which were then randomly assigned to imatinib-treated (5 mice, 10 transplants) and vehicle-treated (6 mice, 12 transplants) groups. At harvest, 7 weeks post-transplantation, CDXs were detected at 11/12 control transplant sites. The CDXs consisted in reddish-brown, flat patches of 4–6 mm in diameter that, as the above-described PDXs, comprised a vasculo-angiogenic network supporting nests of neuroepithelial-like cells (Fig. 7d, upper panels). As in the original PGLs, these cells harboured hyperplastic and swollen mitochondria, not present in the endothelial cells (Online Resource 2, Fig. S4b). In contrast, only 2 CDXs were identified at 10 heterotransplant sites of the imatinib-treated mice (*P* = 0.0015 by two-tailed Fisher exact test). These contained only disorganized autophagic or apoptotic cells, with no signs of an ongoing vasculogenic process (Fig. 7d, lower panels). The in vivo evidence of severe autophagy was consistent with the upregulation of Beclin 1, previously observed in the in vitro treated PTJ64i cells. Standard macroscopic and microscopic analysis of the thoracoabdominal organs did not reveal signs of imatinib toxicity (data not shown). A trend of body weight decrease (average 10%) was noted in the treated group of mice during the first 20 days of treatment, when the mice were injected with imatinib at 50 mg/Kg. Weight was regained in the following 20 days, when the imatinib dose was lowered to one-third (16.6 mg/Kg).

## Discussion

We show here that PGLs are biphasic vascular and neural tumors sustained by stem-like mesenchymal cells primed for bipotent vascular and neural differentiation. These tumors appear to originate through an endogenous developmental program, schematically recapitulated in Fig. S9 (Online Resource 2), where the spatiotemporally coordinated emergence of the two interdependent tissue compartments, both of neoplastic origin, leads to the organization of the neoplastic microenvironment and to predictable mitochondrial changes. This program appears to be independent of the *SDHx* carrier status of the patients, and impacts on preventive and therapeutic interventions, which should be primarily directed against vasculoangiogenesis [22, 28], an early and fundamental phase of PGL tissue organization.

Our conclusions are based on convergent data from *SDHx*-characterized ex vivo, in vitro and in vivo models, including original PGLs, derived cell cultures, cell- and patient-derived xenografts (CDXs and PDXs). These integrative models shed light on the nature of the PGL-initiating cells and on the initial phases of PGL growth.

The PGL histogenetic sequence contrasts with the concept that PGL is primarily a neural tumour [71] and, in general, with the notion that tumour-derived vasculogenesis (vasculogenic mimicry) occurs late in the neoplastic progression [8, 23, 40]. However, it has been shown that primary neoplastic vasculogenesis characterizes the hemangioblastomas associated with von Hippel-Lindau disease, a syndrome that may include PGL [79]. Furthermore, tumour-derived blood vessels were recently and independently reported in PDXs obtained from trunk PGLs and pheochromocytomas [53]. On the other hand, the interconvertibility of mesenchymal stem cells with glial cells has been demonstrated in experimental models [36].

Vasculo-angiogenesis is negatively controlled by miRNAs belonging to the miR-200 and miR-34 families, that antagonize the EMT and promote neural differentiation [25, 48, 50, 70, 77]. We previouly reported that the miR-200a,b,c and -34b,c, decreased in PGLs relative to parasympathetic nerve, control the proliferation and survival of PGL cells [7]. Here we show that these miRs directly and/or indirectly control ZEB1 and PDGFRA in the context of PGL cells, as we previously demonstrated for NOTCH1 [7]. NOTCH1, ZEB1 and PDGFRA are key mediators of vasculo-angiogenesis and radio/chemoresistance [1, 34, 58, 68, 70, 77, 78]. This implicates miRNA dysregulation in PGL biology and aggressiveness.

Regardless of germline *SDHx* mutation status, neuroepithelial differentiation within the PGLs and the PDXs/CDXs was associated with severe mitochondrial dysfunction, implying loss of OXPHOS activity and glycolytic dependence [52, 69, 73]. The mitochondrial alterations were not present in the in vitro cultured PGL stem-like mesenchymal cells, but were acquired in vivo, during PDX/CDX formation in the neuroepithelial cell differentiation context. In this regard it is known that transformed mesenchymal cells, due to their motility and energetic requirements, mainly rely on oxidative phosphorylation [20], whereas tight clusters of neuroepithelial cells, obviously immotile and vulnerable to hypoxic stress due to crowding, must decrease their dependence on OXPHOS, which is compensated by increasing glycolysis [12, 29].

In conclusion, the stem-like mesenchymal component of PGL self-determines its microenvironment, its differentiation and its metabolism according to an endogenous developmental program that may reflect that responsible for physiological paragangliar organogenesis [15, 16]. The biological aggressiveness of the tumour conforms to the inherent tissue regenerative capacities of the multipotent stem-like PGL cells, activated by ischemia and enhanced by resilient embryonic vasculo-angiogenic mechanisms. All this should confer resistance to radio/chemotherapy and standard antiangiogenic therapy [2, 5, 54, 66], and could account for PGL recurrence after treatments that induce ischemic necrosis, such as embolization [61]. However, the fundamental dependence of PGLs on a vasculo-angiogenic phase may provide a targetable Achilles’ heel [17, 43].

## Electronic supplementary material

Below is the link to the electronic supplementary material.

Supplementary material 1 (PDF 631 kb)

Supplementary material 2 (PDF 19777 kb)
