# Supplementary material for: Paragangliomas arise through an autonomous vasculo-angio-neurogenic program inhibited by imatinib
Source: Acta Neuropathol. 2018 Jan 5;135(5):779–98. doi: 10.1007/s00401-017-1799-2 (PMC5904229; doi:10.1007/s00401-017-1799-2)
Supplement: Supplementary file 1 — Supplementary material 1 (PDF 631 kb) [file 401_2017_1799_MOESM1_ESM.pdf]

Online Resource #1

Paragangliomas arise through an autonomous vasculo-angio-neurogenic program inhibited by imatinib

Fabio Verginelli[1,2‡], Silvia Perconti[1,3‡], Simone Vespa[1,3], Francesca Schiavi[4], Sampath Chandra Prasat[5], Paola Lanuti[1], Alessandro Cama[2], Lorenzo Tramontana[6], Diana Liberata Esposito[1,3], Simone Guarnieri[1], Artenca Sheu[1,3], Mattia Russel Pantalone[1,3], Rosalba Florio[2], Annalisa Morgano[1], Cosmo Rossi[1], Giuseppina Bologna[1], Marco Marchisio[1], Andrea D'Argenio[1], Elisa Taschin[4], Rosa Visone[1,3], Giuseppe Opocher[4], Angelo Veronese[1,3], Carlo T. Paties[7], Vinalogu K. Rajasekhar[8], Cecilia Söderberg-Nauclér[9], Mario Sanna[5], Lavinia Vittoria Lotti[6], Renato Mariani-Costantini[1,3*]

[1]Center of Aging Science and Translational Medicine (CeSI-MeT), *Gabriele d'Annunzio* University, Via Luigi Polacchi 11, 66100 Chieti, Italy.
[2]Department of Pharmacy, *Gabriele d'Annunzio* University, Via dei Vestini 31, 66100 Chieti, Italy.
[3]Department of Medical, Oral and Biotechnological Sciences, *Gabriele d'Annunzio* University, Via dei Vestini 31, 66100 Chieti, Italy.
[4]Familial Cancer Clinic and Oncoendocrinology, Veneto Institute of Oncology, IRCCS, Padua, Italy.
[5]Otology and Skull Base Unit, *Gruppo Otologico Piacenza-Roma*, Via Antonio Emmanueli, 42, 29121 Piacenza, Italy
[6]Department of Experimental Medicine, *Sapienza* University of Rome, Viale Regina Elena 324, 00161 Rome, Italy.
[7]Department of Oncology-Hematology, Service of Anatomic Pathology, *Guglielmo da Saliceto* Hospital, Via Taverna 49, 29100 Piacenza, Italy.
[8]Department of Surgery, Memorial Sloan Kettering Cancer Center, New York, New York 10065, USA.
[9]Department of Medicine, Exp Cardiovascular Research Unit and Department of Neurology, Center for Molecular Medicine, Karolinska Institute, Solnavägen 1, 171 77 Solna, Stockholm, Sweden.

[‡]: Equal contribution.

*Corresponding author:
Professor Renato Mariani-Costantini, MD,
Laboratory of General Pathology, Center of Aging Science and Translational Medicine (CeSI-MeT), *Gabriele d'Annunzio* University, Via Luigi Polacchi 11, 66100 Chieti, Italy.
ORCID ID: orcid.org/0000-0002-4440-1848
email address: rmc@unich.it
Tel. +393492914375, Fax +390871541495

Table S1

List of the 77 paraganglioma cases included in the study with selected characteristics of the patients, including sex, age at surgery (for the tumor analyzed here), tumor localization, germline *SDHA, SDHB*, *SDHC*, *SDHD* and *SDHAF2* mutation status, type and effect of the mutation, if present, and SDHB protein immunostaining

Case	Sex/Age at surgery	PGL localization	*SDHx* carrier status	Predicted effect on protein	Mutation type	SDHB IHC
PTJ1[a,c]	F/25	left tympano-jugular	*Noncarrier*			-
PTJ2	M/59	right tympano-jugular	*Noncarrier*			++
PTJ4[c]	M/42	right tympano-jugular	*Noncarrier*			+
PC5[c]	F/42	right carotid body	*SDHD c.445_448dupATCT*	p.(Cys150Tyrfs*42)	small insertion	-
	"	left vagal	"	"	"	-
PTJ6	F/52	right tympano-jugular	*Noncarrier*			++
PC7[c]	F/42	right carotid body	*SDHA c.63+1G>A*[d]	p.?	splicing substitution	-
PTJ8	M/31	right tympano-jugular	*SDHB c.(286+1_287-1)_(*159_?)del*[d]	p.?	gross deletion	-
PT111	M/57	left tympanic	*Noncarrier*			NA
PTJ12	F/74	left tympano-jugular	*Noncarrier*			NA
PTJ13	M/58	right tympano-jugular	*Noncarrier*			+
PT19	F/74	right tympanic	*Noncarrier*			NA
PT20	M/51	right tympanic	*Noncarrier*			++
PTJ21	M/34	right tympano-jugular	*SDHB c.574dupT*[d]	p.(Cys192Leufs*2)	small insertion	-
PTJ34[b,c]	F/25	left tympano-jugular	*SDHB c.778G>C*	p.(Gly260Arg)	missense	-
PT36	F/60	right tympanic	*SDHB c.725G>A*	p.(Arg242His)	missense	-
PTJ37	F/63	right tympano-jugular	*Noncarrier*			++
PTJ43	F/68	right tympano-jugular	*Noncarrier*			+
PT44	F/51	right tympanic	*Noncarrier*			+
PTJ45	M/35	left tympano-jugular	*SDHB c.(423+1_424-1)_(540+1_541-1)del*	p.?	gross deletion	+
PTJ53	F/40	left tympano-jugular	*Noncarrier*			++
PTJ54[c]	M/34	left tympano-jugular	*SDHD c.445_448dupATCT*	p.(Cys150Tyrfs*42)	small insertion	-
PTJ57	F/41	left tympano-jugular	*Noncarrier*			+
PTJ58	M/43	right tympano-jugular	*SDHC c.241+1G>A*[d]	p.?	splicing substitution	++
PTJ59	F/53	left tympano-jugular	*Noncarrier*			+
PT60	F/45	right tympanic	*Noncarrier*			++
PTJ62	F/58	right tympano-jugular	*Noncarrier*			++
PT63	F/63	right tympanic	*Noncarrier*			+
PTJ64	M/33	right tympano-jugular	*SDHC c.43C>T*	p.(Arg15*)	nonsense	+
PV65	M/35	left vagal	*SDHB c.778G>C*	p.(Gly260Arg)	missense	-
PTJ66	F/59	right tympano-jugular	*Noncarrier*			++
PTJ67	F/39	left tympano-jugular	*Noncarrier*			++
PTJ68	M/76	left tympano-jugular	*Noncarrier*			++
PT69	F/43	right tympanic	*SDHB c.778G>C*	p.(Gly260Arg)	missense	-
PT70	M/37	left tiympanic	*Noncarrier*			++
PTJ71	F/52	left tympano-jugular	*Noncarrier*			++
PT72	F/43	left timpanic	*SDHB c.218T>A*[d]	p.(Leu73*)	nonsense	+
PTJ73	M/44	right tympano-jugular	*Noncarrier*			-
PT74	F/57	right tympanic	*Noncarrier*			++
PTJ76	M/51	left tympano-jugular	*SDHB c.423+1G>T*[d]	p.?	splicing substitution	-
PTJ77	F/63	right tympano-jugular	*Noncarrier*			-
PTJ78	F/55	right tympano-jugular	*Noncarrier*			+
PTJ79	M/36	tympano-jugular	*Noncarrier*			+
PTJ80	M/55	right tympano-jugular	*Noncarrier*			++
PT81	M/51	right tympanic	*Noncarrier*			++
PT82	F/52	left tympanic	*Noncarrier*			++
PT83	F/70	left tympanic	*Noncarrier*			++
PTJ84	M/46	left tympano-jugular	*Noncarrier*			-
PT85	M/53	right tympanic	*Noncarrier*			++
PTJ86[a]	M/15	left tympano-jugular	*SDHD c.27delC*[d]	p.(Val10Phefs*5)	small deletion	++
PV87	F/43	right vagal	*Noncarrier*			++
PV88	F/48	left vagal	*Noncarrier*			-
PTJ89	F/49	right tympano-jugular	*Noncarrier*			++
PT90	F/58	right tympanic	*Noncarrier*			++
PC91	F/56	left carotid body	*SDHA c.1607delG*[d]	p.(Cys536Leufs*11)	small deletion	-
PTJ92	M/51	right tympano-jugular	*SDHC c.(179+1_180-1)_(*2318_?)del*	p.?	gross deletion	++
PTJ93	M/44	left tympano-jugular	*SDHC c.43C>T*	p.(Arg15*)	nonsense	++
PC94	F/37	left carotid body	*Noncarrier*			NA

Continued

Case	Sex/Age at surgery	PGL localization	*SDHx* carrier status	Predicted effect on protein	Mutation type	SDHB IHC
PTJ95	F/45	right tympano-jugular	*SDHC c.(78+1_180-1)_(241+1_242-1)dup*	p.?	gross duplication	+
PTJ96	F/56	right tympano-jugular	*Noncarrier*			++
PT97	F/73	right tympanic	*Noncarrier*			++
PC98[c]	F/44	right carotid body	*Noncarrier*			++
PTJ99	F/45	left tympano-jugular	*SDHC c.377A>G*[e]	p.(Tyr126Cys)	missense	-
PV100	M/46	left vagal	*SDHD c.242C>T*	p.(Pro81Leu)	missense	NA
PTJ101	M/37	right tympano-jugular	*Noncarrier*			NA
PV102	F/40	right vagal	*SDHA c.889C>T*[d]	p.(Pro297Ser)	missense	+
PTJ103	M/38	left tympano-jugular	*SDHAF2 c.261-2A>T*[d]	p.?	splicing substitution	NA
PT104	F/55	right tympanic	*Noncarrier*			NA
PV105	F/32	left vagal	*SDHB c.(?_-151)_(72+1_73-1)del*	p.?	gross deletion	-
PTJ106	M/56	left tympano-jugular	*Noncarrier*			++
PT107	F/44	left tympanic	*Noncarrier*			++
PTJ114	F/50	left tympano-jugular	*NA*			NA
PTJ118	F/13	right tympano-jugular	*NA*			NA
PTJ119	M/40	right tympano-jugular	*NA*			NA
PC120	F/43	left carotid body	*NA*			NA
PTJ122[c]	M/26	right tympano-jugular	*NA*			NA
PTJ123	F/51	left tympano-jugular	*NA*			NA

NA: not available; **[a]:** documented paraganglioma family history; **[b]:** metastases in 4 out 17 lymph nodes; **[c]:** history of multiple/recurrent disease; **[d]:** novel mutation; [e]: variant present in ClinVar or LOVD3.0

Table S1

All the patients were admitted for elective surgery without any previous therapy. Clinical genetic testing for germline *SDHx* mutations, including large deletions and rearrangements, was performed as described [1] on blood for a subset of 70 patients recruited from 11-2009 to 3-2016. *SDHx* mutational data for the 7 cases recruited from 5-2016 to 6-2017 are at present not available. One case (PTJ34) had lymph node metastases, one case had two independent tumors (5PC/PV), 9 patients presented with paraganglioma recurrency. Preoperative urinary and plasma catecholamines were negative, in agreement with the mostly parasympathetic (non secretory) origin of head and neck paragangliomas [2, 3]. Only two patients (PTJ1 and PTJ86) reported a family history of paraganglioma, and none reported synchronous or metachronous thoracoabdominal paragangliomas or pheochromocytomas. Sites of tumor origin, encoded in the case acronyms, include carotid body (paraganglioma, carotid body: PC, 6 cases), cervical portion of the vagus nerve (paraganglioma, vagus nerve: PV, 7 cases), tympano-jugular region (paraganglioma, tympano-jugular: PTJ, 45 cases), tympanic nerve (paraganglioma, tympanic: PT, 19 cases). The median age at surgery for the series of 77 cases was 47.6 years (13-76 years), 30 patients (39.5%) were males, 46 patients (60.5%) females; median age at surgery for males was 47.8 (15-76), for females 47.6 (13-74). Overall, germline *SDHx* mutations were detected in 24/70 (34.3%) examined patients (5PC and 5PV are metachronous tumors of a single patient). Notably 10/24 mutations (41.6%) appear to be novel.

Table S2

Sites of tumor origin, age, sex, *SDHx* germline mutation status and SDHB immunohistochemistry (IHC) in the subset of 71 independent paragangliomas from 70 patients analyzed for germline *SDHx* mutation status

PGL site[a] (#, %)	Mean age (range)	Sex (F-M)	*SDHx* germline mutations						# carriers/total (%)	SDHB loss (71 tumors[c])
			SDHB	*SDHC*	*SDHD*	*SDHA*	*SDHAF2*	*Noncarriers*		
**T (19; 26,8%)**	54.6 (37-74)	14F-5M	3					16	3/19 (15.8%)	3/16 (18.8%; 3 NA)
**TJ (40; 56,3%)**	47 (15-76)	19F-21M	5	6	2		1	26	14/40 (35%)	9/37 (24.3%; 3 NA)
**C (5; 7%)**[b]	46.2 (37-56)	5F			1	2		2	3/5 (60%)	3/4 (75%; 1 NA)
**V (7; 9,9%)**[b]	42.3 (32-52)	5F-2M	2		2	1		2	5/7 (71.4%)	4/6 (66.7%; 1 NA)
**Total (71)**	48.5 (15-76)	43F-28M	10	6	5	3	1	46	25/71 (35.2%)	19/63 (30.2%; 8 NA)

NA: Not available; **[a]:** T, tympanic; TJ, tympano-jugular; C, carotid body; V, vagal; **[b]:** one case had two independent tumors (PC/PV5, carotid body and vagal paragangliomas respectively); **[c]:** eight cases could not be analyzed for SDHB IHC because the paraffin embedded samples were affected by embolization. Overall the comparatively high frequencies of tympanic and tympano-jugular paragangliomas reflects recruitment at a specialized skull base surgery center.

Table S3

Age, sex and tumor-associated SDHB protein loss in paraganglioma cases from *SDHx* mutation carriers and noncarriers (70 patients, 71 tumors)

***SDHx* status**	**Patients (%)**	**Mean age (min-max)**		**Sex (F-M)**	**SDHB loss (71 tumors[a])**	
SDHB	10/70 (14.3%)	38.9 (25-60)		5F-5M	8/10 (80%)	
SDHC	6/70 (8.6%)	43.5 (33-51)		2F-4M	1/6 (1,66%)	
SDHD	4/70 (5.7%)	36.7 (15-52)		1F-3M	3/4 (75%; 1 NA)	
SDHA	3/70 (4.3%)	46 (40-56)		3F	2/3 (66.66%)	
SDHAF2	1/70 (1.4%)	38		1M	NA	
***SDHx* carriers**	**24/70 (34.3%)**	**40,5 (15-60)**	**$P < 0.0001$**	**12F-13M**	**14/23[(*)] (60.9%; 2 NA)**	**$P = 0.0001$**
**Noncarriers**	**46/70 (65.7%)**	**52.6 (25-76)**		**31F-15M**	**5/40[(*)] (12,5%; 6 NA)**	

NA: not available; **[a]:** Seventy one paragangliomas were evaluated, as an *SDHD* carrier presented with two metachronous paragangliomas (PC/PV5). Eight paragangliomas could not be evaluated for SDHB immunostaining because the paraffin-embedded tissue was damaged by embolization.

Particular features of our case series include higher frequency of mutations in *SDHB* and not in *SDHD* [2] and relatively high frequency of mutations in *SDHA*, the latter also noted in a recent independent study [4]. These features might reflect the rarity of familial PGL/PC history (see Table S1) [2, 3]. Statistical differences were calculated using 2-tailed unpaired t- test (age) or 2-tailed Fisher exact test (SDHB loss).

Table S4

Flow cytometric analysis of dissociated total paraganglioma cells reveals cell populations positive for the mesenchymal surface markers CD73, CD90 and CD105

**Case acronym**	**CD73+ (%)**	**CD90+ (%)**	**CD105 (%)**	***CD73+/ CD105+ (%)***
**PC120**	**13.3**	**42.8**	**41.4**	*12.4*
**PTJ121[1]**	**21.2**	**3.3**	**40.1**	*17.3*
**PTJ121[2]**	**24.4**	**2.2**	**47.7**	*22.1*
**PTJ122**	**67.0**	**6.8**	**2.2**	*2.2*
**PTJ123**	**64.6**	**10.5**	**2.1**	*2.1*

The columns in bold report the total fractions of dissociated cells positive for CD73, CD90, and CD105, the last column in italics reports the cell fractions positive for both CD73 and CD105 (CD73+/CD105+). PTJ121[1] and PTJ121[2] are distinct samples from a single tumor. Mutation status for these cases is currently not available.

Table S5

Flow cytometric analysis of dissociated total paraganglioma cells shows that the CD133/CD44-positive subset contains variable fractions of cells with CD34++/CD45- phenotype

**Case**	***Mut*/SDHB IHC**	**CD34++/CD45- (%)**	**CD133+/CD44+ (%)**	**CD34++/CD45/CD133+/CD44+ (%)**
**PTJ99**	*SDHC*/SDHB-	1	82.3	0.3
**PV100**	*SDHD*/NA	0.1	79.1	0.1
**PTJ101**	*Noncarrier*/NA	1.4	65	0.8
**PTJ103**	*SDHAF2*/NA	4.1	5.5	0
**PTJ106**	*Noncarrier*/SDHB+	75.1	76.4	89
**PTJ109**	*NA*/NA	23	83.1	25.5
**PTJ114**	*NA*/NA	72.6	19.4	53.5
**PTJ119**	*NA*/NA	62	0.2	14.3

NA: not available.

The first two columns report the total fractions of cells positive for surface CD34 (CD34++/CD45-) and for CD133/CD44 (CD133+/CD44+). The last column reports the cell fractions with CD34++/CD45 phenotype found within the CD133+/CD44+ subset. Variability is expected, because of the heterogeneous cellular composition of the tumors and of preoperative embolization.

Table S6

Flow cytometric analysis of dissociated total paraganglioma cells shows that the NCAM- and the GFAP-positive subsets contain variable fractions of cells with CD34++/CD45- phenotype

Case	Mut/IHC	CD34++/CD45- (%)	NCAM+ (%)	*CD34++/CD45/NCAM+ (%)*	GFAP+ (%)	*CD34++/CD45/GFAP+ (%)*
**PTJ64**	*SDHC*/SDHB+	**2**	**25.4**	23.3	**1**	*3*
**PV65**[a]	*SDHB*/SDHB-	**7.5**	**61.6**	*0.4*	**7.4**	*0.4*
**PTJ67**	*Noncarrier*/SDHB-	**0.2**	**0.03**	0.3	**0.13**	*1*
**PTJ78**	*Noncarrier*/SDHB+	**22.6**	**41.6**	*1.7*	**NA**	*NA*
**PTJ79**	*Noncarrier*/SDHB+	**34.4**	**91.5**	97.0	**NA**	*NA*
**PTJ80**	*Noncarrier*/SDHB+	**12.8**	**23.5**	*25.6*	**NA**	*NA*
**PV87**[a]	*Noncarrier*/SDHB+	**36.6**	**39.7**	35.0	**96.2**	*92.5*
**PV88**[a]	*Noncarrier*/SDHB-	**0.2**	**70**	*0*	**7**	*0*
**PTJ89**	*Noncarrier*/SDHB+	**0**	**0.4**	0	**NA**	*NA*[a]

NA: not available; [a]**:** also characterized for GFAP/NCAM double positivity (0.2%, 40.7%, 6.6% respectively). Furthermore, in PV87 and PV88 a fraction of the GFAP/NCAM double positive cells (14.3% and 22% respectively) was inside the CD34++ population.

The columns in bold report the total fractions of dissociated cells positive for surface CD34 (CD34++/CD45-), surface NCAM and, when available, intracellular GFAP (variability may reflect tissue embolization and heterogeneous vascular and neural tumor tissue composition). The columns in italics report the fractions of NCAM+ or GFAP+ cells found inside the CD34++ brilliant population.

Table S7

Flow cytometry of paraganglioma cell cultures grown in adhesion

Case	*Mut*/IHC	CD73	CD90	CD105	CD133	SOX2	Nestin	PDGFRA	GFAP	NCAM	CD34
**PTJ64p**	*SDHC*/SDHB+	++	+++	++	NA	+	NA	NA	++	++[c]	-
**PTJ64i**[a]		+++	+++	++	-	+++	+++	+	+++	+[d]	++
**PTJ67p**	*Noncarrier*/SDHB-	NA	NA	NA	NA	NA	++	NA	+	-	NA
**PTJ78p**	*Noncarrier*/SDHB+	NA	++	NA	NA	NA	NA	NA	++	-	-
**PTJ79p**	*Noncarrier*/SDHB+	++	+[e]	++	NA	+++	++	NA	+++	-	-
**PTJ80p**	*Noncarrier*/SDHB+	++	+++	NA	NA	+++	+++	++	-	-	-
**PTJ84p**	*Noncarrier*/SDHB-	++	+++	++	NA	++	++	++	-	+	-
**PTJ86p**	*SDHD*/SDHB+	+++	+++	NA	NA	+	+++	++	-	-	-
**PTJ86i**[a]		+++	+++	++	-	+++	+++	+	+++	+	++
**PV87p**	*Noncarrier*/SDHB+	+	++	NA	NA	++	++	++	-	-	-
**PTJ89p**	*Noncarrier*/SDHB+	NA	NA	++	NA	NA	NA	NA	++	+	NA
**PTJ89i**[a]		+++	++	++	-	++	+++	+	+++	-	+
**PT90p**	*Noncarrier*/SDHB+	NA	++	NA	NA	NA	++	NA	-	-	++
**PC94p**	*Noncarrier*/NA	NA	NA	NA	NA	++[f]	++	NA	++	-	NA
**PTJ98i**[b]	*Noncarrier*/SDHB+	+++	++	++	-	+++	+++	+	+++	+	+
**PV105p**	*SDHB*/SDHB-	++	++	++	-	++	+	+	-	-	-
**PTJ106p**	*Noncarrier*/SDHB+	+++	+++	NA	-	++	++	-	-	-	+
**PTJ114p**	*NA*/NA	++	+++	++	-	+++	+++	++	+++	-	+
**PTJ118p**	*NA*/NA	NA	NA	NA	-	++	++	NA	NA	NA	-
**PTJ119p**	*NA*/NA	++	+++	++	-	++	++	+	+++	-	-
**PC120p**	*NA*/NA	+++	++	++	-	++	++	NA	-	-	+
**PTJ121p**	*NA*/NA	++	++	++	-	++	++	-	++	+	+

NA: Not assessed [a]**:** Immortalized with SV40 and hTERT**;** [b]**:** Immortalized with SV40; [c]**:** Positive cells =12.4%; [d]**:** Positive cells = 1.4%; [e]**:** Positive cells = 30%; [f]**:** Positive cells = 57.6%.

Twenty-two (22) cultures from 19 paragangliomas (18 primary and 4 immortalized), were analyzed by flow cytometry for putatively mesenchymal (CD73, CD90, CD105), stem cell (CD133, SOX2, nestin), developmental (PDGFRA), glial/neural (GFAP, NCAM) and endothelial (CD34++/CD45-) markers. Primary cells were analysed within passage 11, immortalized cells at passages 25 to 43. Matched primary and immortalized cultures are identified by shared background color (light blue). Given the prevalently homogeneous positivity of the cells, results are presented according to a semiquantitative scale based on the mean fluorescence intensity (MFI) ratio (MFIRs), i.e., the ratio between the MFI of the cells stained with the specific antibody and the MFI of the respective control, as follows: MFIR ≤ 2.5 = negative (-); MFIR 2.5–10 = weakly positive (+); MFIR 10 -100 = moderately positive (++); MFI > 100 =

strongly positive (+++). Bimodal antigen distribution was observed only in 4 instances, for which % of positive cells are provided in the notes. The Mut/SDHB IHC column details if a germline *SDHx* mutation (Mut) was detected (affected gene) or not and if SDHB immunohistochemistry was positive (+) or negative (-) in the tumor from which the culture was developed. As evident here, germline *SDH*x mutation status did not affect the phenotype of the cell cultures. Low cell numbers precluded systematic testing of the primary cultures for all markers.

Table S8
Xenograft formation from patient-derived paraganglioma samples transplanted into NSG mice

Patient code (sex/age)	*SDHx* carrier status[a]	Graft site(s)	Growth time (months)	Grafted PGL sample(s) (n°)	PDX formation (n°)[b]
**PV65 (M/35)**	*SDHB*	Flank rt	5	1	1
**PTJ79 (M/36)**	*Noncarrier*	Flank rt	7	1	1
**PV87 (F/43)**	*Noncarrier*	Flanks, bilateral	5	2	2
**PV88 (F/48)**	*Noncarrier*	Flanks, bilateral	10	2	2
**PTJ89 (F/49)**	*Noncarrier*	Flank rt	8	1	1
**PTJ90 (F/58)**	*Noncarrier*	Flank rt	8	1	1
**PTJ91 (F/51)**	*SDHC*	Flanks, bilateral	8	4	4
**PTJ93 (M/44)**	*SDHC*	Neck, bilateral	6	4	3
**PTJ96 (F/56)**	*Noncarrier*	Neck, bilateral	5	2	2
**PC98 (F/44)**	*Noncarrier*	Neck, bilateral	6	4	4
**PTJ101 (M/37)**	*Noncarrier*	Flanks, bilateral	5	4	4
**PTJ103 (M/38)**	*SDHAF2*	Flanks posterior and anterior, bilateral	5	8	5
**PV105 (F/32)**	*SDHB*	Flanks and neck, bilateral	4.5	16	12
**PTJ106 (M/56)**	*Noncarrier*	Flanks and neck, bilateral	4.5	6	4
**PTJ114 (F/50)**	*NA*	Flanks, bilateral	6	30	30
**PC120 (F/43)**	*NA*	Flanks posterior and anterior, bilateral	3 weeks	4	4

NA: not available; **[a]:** gene specified for cases with identified *SDHx* mutation; **[b]:** PDX formation was always confimed by epon-embedded semithin section light microscopy, electron microscopy and/or frozen section ApoTome immunofluorescence.

Relevant *SDHx* mutation status of the donor patient, graft sites, growth times, number of transplanted paraganglioma (PGL) samples and xenograft formation are indicated. PDX formation was obtained from 80/90 transplanted paraganglioma samples (89%). No significant differences in engraftment rates were observed between samples derived from *SDHx* mutation carriers versus noncarriers. The rates of engraftment were not affected by the anatomic localizations of the original tumors. Control normal tissue transplants (3 from abdominal skin of paraganglioma patients, not listed in the Table) underwent regression and calcification.

Supplemental references

1. von Dobschuetz E, Leijon H, Schalin-Jantti C, Schiavi F, Brauckhoff M, Peczkowska M, et al (2015) A registry-based study of thyroid paraganglioma: histological and genetic characteristics. Endocrine-related cancer 22:191-204. Doi 10.1530/ERC-14-0558
2. Pacak K, Wimalawansa SJ (2015) Pheochromocytoma and paraganglioma. Endocr Pract 21: 406-412. Doi 10.4158/EP14481.RA
3. Taieb D, Kaliski A, Boedeker CC, Martucci V, Fojo T, Adler JR, Jr., Pacak K (2014) Current approaches and recent developments in the management of head and neck paragangliomas. Endocr Rev 35: 795-819. Doi 10.1210/er.2014-1026
4. Bausch B, Schiavi F, Ni Y, Welander J, Patocs A, Ngeow J et al (2017) Clinical Characterization of the Pheochromocytoma and Paraganglioma Susceptibility Genes SDHA, TMEM127, MAX, and SDHAF2 for Gene-Informed Prevention. JAMA Oncol. 3:1204-1212. doi: 10.1001/jamaoncol.2017.0223.
