# Supplementary material for: Paragangliomas arise through an autonomous vasculo-angio-neurogenic program inhibited by imatinib
Source: Acta Neuropathol. 2018 Jan 5;135(5):779–98. doi: 10.1007/s00401-017-1799-2 (PMC5904229; doi:10.1007/s00401-017-1799-2)
Supplement: Supplementary file 2 — Supplementary material 2 (PDF 19777 kb) [file 401_2017_1799_MOESM2_ESM.pdf]

Online Resource #2

Paragangliomas arise through an autonomous vasculo-angio-neurogenic program inhibited by imatinib

Fabio Verginelli[1,2‡], Silvia Perconti[1,3‡], Simone Vespa[1,3], Francesca Schiavi[4], Sampath Chandra Prasat[5], Paola Lanuti[1], Alessandro Cama[2], Lorenzo Tramontana[6], Diana Liberata Esposito[1,3], Simone Guarnieri[1], Artenca Sheu[1,3], Mattia Russel Pantalone[1,3], Rosalba Florio[2], Annalisa Morgano[1], Cosmo Rossi[1], Giuseppina Bologna[1], Marco Marchisio[1], Andrea D'Argenio[1], Elisa Taschin[4], Rosa Visone[1,3], Giuseppe Opocher[4], Angelo Veronese[1,3], Carlo T. Paties[7], Vinalogu K. Rajasekhar[8], Cecilia Söderberg-Nauclér[9], Mario Sanna[5], Lavinia Vittoria Lotti[6], Renato Mariani-Costantini[1,3*]

[1]Center of Aging Science and Translational Medicine (CeSI-MeT), *Gabriele d'Annunzio* University, Via Luigi Polacchi 11, 66100 Chieti, Italy.
[2]Department of Pharmacy, *Gabriele d'Annunzio* University, Via dei Vestini 31, 66100 Chieti, Italy.
[3]Department of Medical, Oral and Biotechnological Sciences, *Gabriele d'Annunzio* University, Via dei Vestini 31, 66100 Chieti, Italy.
[4]Familial Cancer Clinic and Oncoendocrinology, Veneto Institute of Oncology, IRCCS, Padua, Italy.
[5]Otology and Skull Base Unit, *Gruppo Otologico Piacenza-Roma*, Via Antonio Emmanueli, 42, 29121 Piacenza, Italy
[6]Department of Experimental Medicine, *Sapienza* University of Rome, Viale Regina Elena 324, 00161 Rome, Italy.
[7]Department of Oncology-Hematology, Service of Anatomic Pathology, *Guglielmo da Saliceto* Hospital, Via Taverna 49, 29100 Piacenza, Italy.
[8]Department of Surgery, Memorial Sloan Kettering Cancer Center, New York, New York 10065, USA.
[9]Department of Medicine, Exp Cardiovascular Research Unit and Department of Neurology, Center for Molecular Medicine, Karolinska Institute, Solnavägen 1, 171 77 Solna, Stockholm, Sweden.

[‡]: Equal contribution.

*Corresponding author:
Professor Renato Mariani-Costantini, MD,
Laboratory of General Pathology, Center of Aging Science and Translational Medicine (CeSI-MeT), *Gabriele d'Annunzio* University, Via Luigi Polacchi 11, 66100 Chieti, Italy.
ORCID ID: orcid.org/0000-0002-4440-1848
email address: rmc@unich.it
Tel. +393492914375, Fax +390871541495

Appendix to Materials and Methods

Immunomorphological studies. Marker proteins and relative antibodies included: HIF2A (Abcam ab199, 1:50); ZEB1 (Millipore ABN285, 1:100, overnight at 4°C); NOTCH1 (Santa Cruz C-20, recognizing full-length NOTCH1 and the NOTCH1 intracellular domain NICD1, 1:50); delta like non-canonical NOTCH ligand 1 (DLK1, Abcam ab119930, 1:50); PDGFRA (Santa Cruz sc-338, 1:50); VEGFR1 (Santa Cruz sc-316, 1:100); VEGFR2 (Santa Cruz sc-504, 1:50); GLUT4 (Novus Biologicals NBP1-49533, 1:200); CD34 (Novocastra NCL-L-END, 1:50); CD31 (Abcam ab28364, 1:50); nestin (Santa Cruz sc-23927, 1:50); NCAM/CD56 (Santa Cruz sc-106, 1:50); S100 (Novocastra, NCL-L-S100p, 1:50); β3-tubulin (β3Tub, Santa Cruz sc-80005, 1:50); chromogranin A (CGA, Santa Cruz sc-271738, 1:50); alpha-smooth muscle actin (SMA, DAKO M851, 1:400); vimentin (Stemgent #09-0077, 1:100); BAX (Millipore MAB4601, 1:100), CD44 (Santa Cruz sc-7297, 1:100); KIT (eBioscience mab ACK2, 4 µg/ml); collagen I (Sigma C2456, 1:100); collagen IV (Chemicon ab748, 1:20). The antibodies used to discriminate human from murine cells included: 1) rat anti-mouse CD31 (PECAM1, BD Pharmingen #550274) mixed 1:1 with rat anti-mouse CD105 (endoglin, BD Pharmingen #550546), both specific for mouse angiogenic cells (each diluted 1:50); 2) anti-human β_2 microglobulin (Santa Cruz sc-13565, 1:50); 3) the anti-mitochondrial antibody 113-1, which identifies a 65 Kd non-glycosylated mt surface protein expressed in human but not in rodent cells (Abcam ab92824, 1:100). The secondary antibodies, all diluted 1:200, included: goat anti-mouse IgG fluorescein isothiocyanate-conjugated (Alexa 488, Lifetech); goat anti-rabbit IgG Texas-Red-conjugated (Jackson ImmunoResearch); goat anti-rat FITC (ICN Pharmaceuticals); CF594 goat anti-mouse IgG, (Biotium); CF488 goat anti-rabbit IgG (Biotium). Nuclei were visualized with 4',6-diamidino-2-phenylindole dihydrochloride (DAPI) (Sigma-Aldrich) or DRAQ5 (Thermo Fisher Scientific).

Cytofluorimetry. Freshly dissociated cells were profiled using a FACSCantoII flow cytometer running with FACSDiVa software (BD) and settings as in Lachmann et al. [1]. The following markers were evaluated: CD133 (Miltenyi #130-098-829), CD44 (BD #555479), NCAM/CD56 (BD #557747), GFAP (BD #561483), CD34 (BD #562577), CD45 (BD #560367), CD90 (BD #555595), CD105 (endoglin, Ancel #326-050), CD73 (BD #550257), Syto16 (TermoFisher #S-7578), 7AAD (BD #559925).

Primary and immortalized cultures were analyzed with the same antibodies as above, plus nestin (BD #561231), PDGFRA (Santa Cruz sc-338) and SOX2 (BD #560301). Dilutions were based on titrations under assay conditions. Instrument performance and data reproducibility were checked using the Cytometer Setup & Tracking Module (BD Biosciences) and validated with Rainbow Beads (BD Biosciences). Compensation was calculated using CompBeads (BD Biosciences) and single-stained samples. Prior to each run the flow cytometer was cleaned according to manufacturer's instructions and the FACSCanto SIT Flush device was routinely used to prevent carryover. Data were analyzed using FACSDiva v 6.1.3 (BD), FACSuite v1.05 (BD) and FlowJo v 8.8.6 (TreeStar, Ashland, OR, USA) softwares. Antibody specificities were assessed using isotype matched controls and, for surface reagents, fluorescence minus one (FMO) controls [2]. Controls also included unstained cells and cells stained with secondary antibody only (when applicable).

MiRNAs. MiRNA real□time quantitative reverse transcription PCRs (qRT□PCRs) were performed with previously reported oligonucleotides [3] on total TRIzol-purified RNA (Invitrogen) quantified with Nanodrop 2000 (Thermo Scientific). MiRNA sequences were from miRBase (http://www.mirbase.org/index.shtml). Stem loop RT primers were designed to include the Universal Probe Library (UPL) #21 sequence binding site (UPL database, Roche Diagnostics). Total RNA (50 ng) was retro-transcribed with the TaqMan Micro-RNA Reverse Transcription Kit (Life Technologies). Reactions were incubated 30 min at 16°C, followed by pulsed RT of 60 cycles at 30°C for 30 s, 42°C for 30 s, and 50°C for 1 s. SH-SY5Y neuroblastoma cells (ATCC; CRL-2266), acquired in 2007 and authenticated in January 2013 using AmpFISTR-Identifiler-Plus kit (Life Technologies), were transfected using Lipofectamine 2000 (Invitrogen) with miRNA mimics precursor (75 ng/10,000 cells) or expression vectors (75 ng/10,000 cells) as negative control (Life Technologies) [3]. Firefly and renilla luciferase activities were measured according to dual luciferase assay protocol (Promega) after 24 h. Lentiviral infection was required in PGL cells because of inefficient liposome transfection. MiRNA-expressing lentiviruses (PMIRH200b-429PA-1, PMIRH34bcPA-1, System Bioscience) were generated using Lentivector-based microRNA precursor constructs (System Biosciences), control lentiviral particles were from System Biosciences (Cod. SBPMIRH000VA1). PGL cells were seeded at 3.6 × 104 cells per well in 12-well plates in complete culture medium and infected at a multiplicity of infection (MOI) of 50. Infections were performed at least in triplicate. To assess effects on PDGFRA expression, the PDGFRA 3'-UTR was amplified in two fragments from DNAs of 3 healthy donors, using primer sets designated PDGFRA200a_3677 PmeI (5'-AGCTTTgtttaaacGCAGAGGTTGAGAGGAGGAC-3')/PDGFRA200a_4288 NotI (5'-AAATATgcggccgcTGTCAGGCTTCTAAATGACCCAA-3') for the F1R1 fragment and PDGFRA200a_5298 PmeI (5'-AGCTTTgtttaaacATTGAACTTTCCCCGTCTCCC-3')/PDGFRA200a_5577 NotI (5'-

AAATATgcggccgcTCACAGCCCATGTTTCATTCAT-3') for the F2R2 fragment. Pooled PCR products were cloned downstream of the firefly luciferase gene in the psiCHECK-2 vector (Promega) using the Rapid DNA Ligation Kit (Roche), competent NZY5α bacterial cells (NZYTech) and the PureLink HiPure Plasmid Maxiprep Kit (Thermo Fisher). Substitutions and/or deletions into the miR-200a binding sites of the PDGFRA 3'UTR were introduced by gBLOCK gene fragment technology (Integrated DNA Technologies). Purchased gBLOCK fragments F1R1-mut (5'-…GAACTTTGTGGACACAGCCTCTTGC…-3'; 5'-…CTATCTTTT------TTAAAGAGA…-3') and F2R2-mut (5'-…TATGCAATT--G-G---CAAGTCTCTGTGTA-----------GAAAAAACA..-3') were cloned into the psiCHECK-2 vector with an intermediate step into the pCR 2.1 Topo-TA vector (Thermofisher). All PDGFRA fragments were verified by Sanger sequencing.

Western blotting. Triplicate whole lysates were prepared from cells scraped from six-well plates and dissolved in lysis buffer (M-PER; Thermo Scientific) with protease (GE Healthcare) and phosphatase (Sigma-Aldrich) inhibitors. After electrophoresis and blotting primary antibodies to PDGFRA (Santa Cruz SC-338), PDGFRB (Santa Cruz, sc-339), ZEB1 (Millipore ABN285), antiphosphotyrosine (Cell Signaling P-Tyr-100), beclin-1 (Cell Signaling #3495), vinculin (Santa Cruz SC5573), and ß-actin (Cell Signaling 4967) were incubated overnight at 4°C. Horseradish peroxidase-conjugated anti-mouse or anti-rabbit antibodies (Bio-Rad) were incubated for 1 h at room temperature and detected using chemiluminescence (Pierce ECL Western Blotting Substrate; Thermo Scientific). Loading was normalized with ß-actin or vinculin. Digitalized signals were quantified in the linear range of the scanner using ImageJ 64 (http://imagejdocu.tudor.lu/).

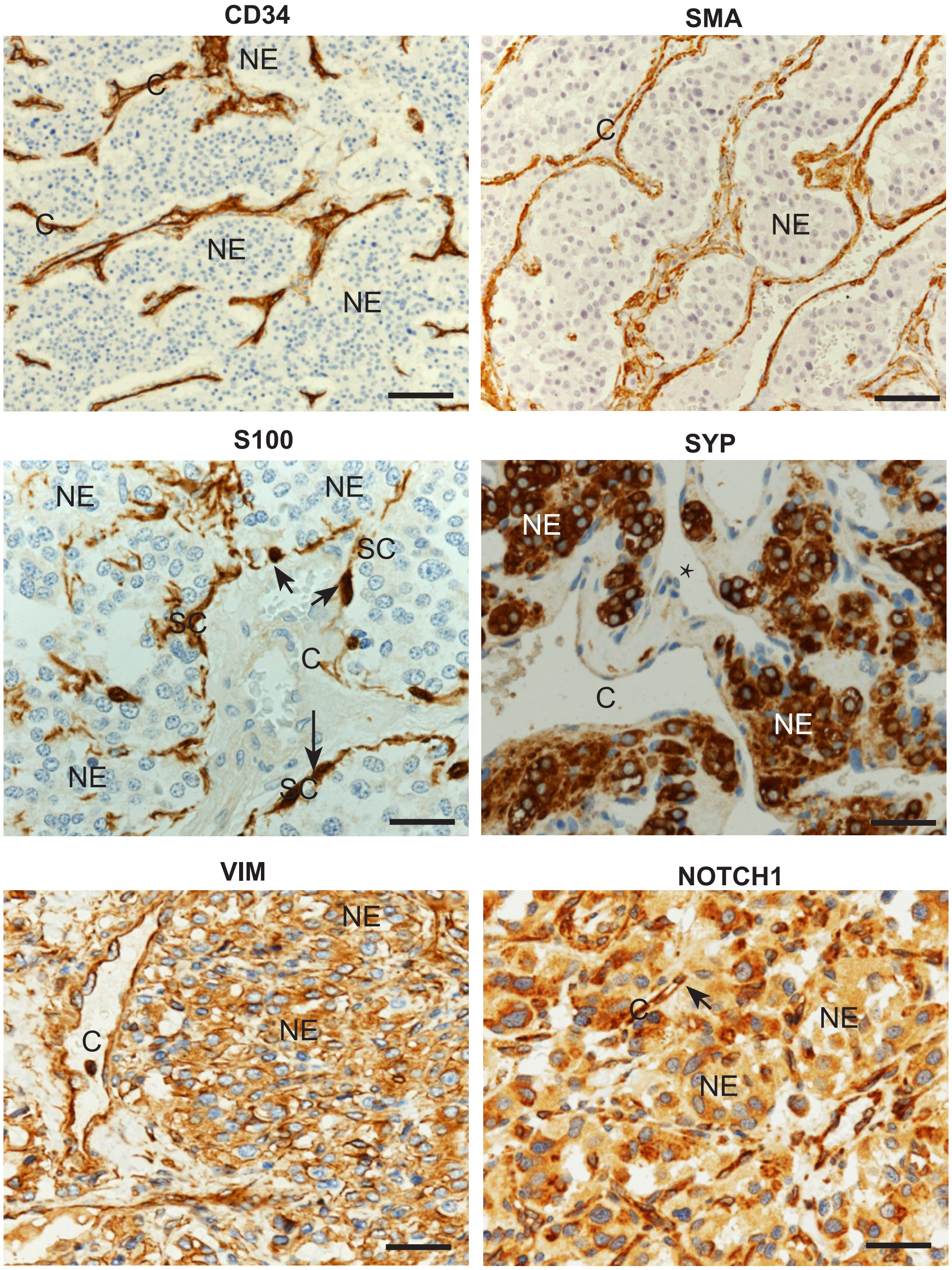

Figure S1
Standard immunohistochemistry shows that the vascular and the neural compartments of paragangliomas can be distinguished using tissue-specific markers but share mesenchymal markers. CD34 and alpha-smooth muscle actin (SMA) stain the vascular tumor network (endothelia and pericytes, respectively), while the interposed neuroepithelial nests ("zellballens", ZB, arrowheads) show only hematoxylin (blue) counterstaining. S100 labels the thin sustentacular cells that circumscribe the zellballens (arrows), while the zellballens (ZB) and the supporting vascular network (*capillary lumen) are negative. Synaptophysin (SYP) strongly labels the zellballens (ZB), while the capillaries (*) are negative. In contrast, Vimentin (VIM) and NOTCH1 are detectable in the zellballens (ZB) and along the vascular and perivascular network (arrows). Bars = 100 µm (CD34, SMA) and 50 µm (S100, SYP, VIM, NOTCH1). Data were confirmed on all the paraganglioma case series (77 cases)

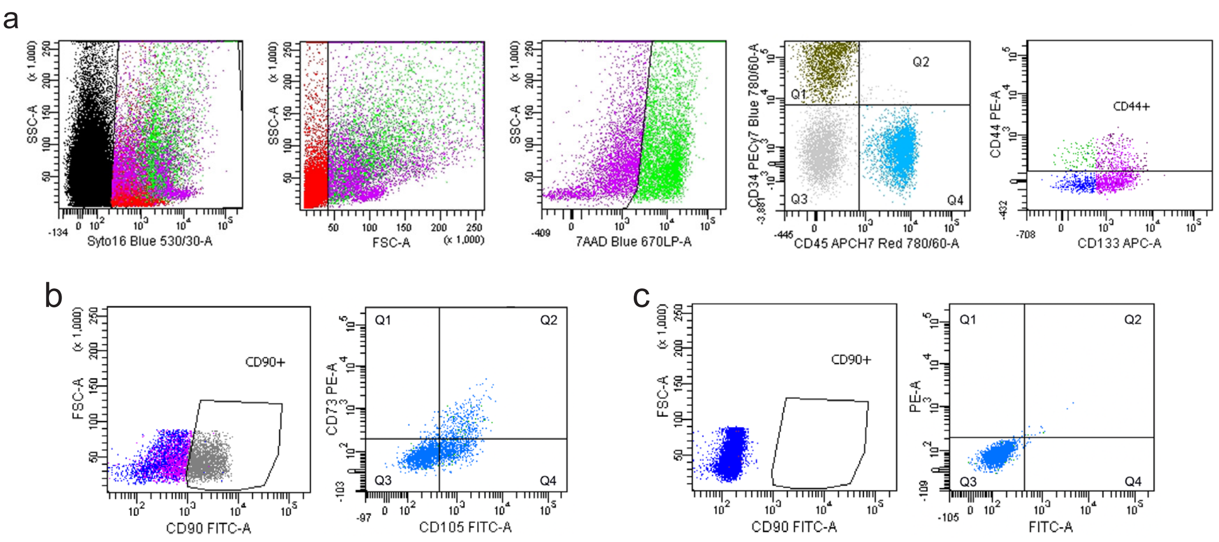

Figure S2

Flow cytometric characterization of dissociated paraganglioma cells. (a) Gating strategy for the identification of cells positive for CD34 and CD44/CD133 in paraganglioma. Cells from dissociated tumor samples were identified on the basis of SYTO 16 green fluorescent nucleic acid stain and first level gated on a forward versus side scatter plot (SSC-A/FSC-A). Dead cells were excluded after analysis of cell viability using 7-Amino Actinomycin D (7-AAD), then live cells were analyzed for surface expression of CD34/CD45 and CD44/CD133. **(b)** Cytofluorimetric identification of cells positive for the surface-related mesenchymal markers CD90, CD105 and CD73 in paraganglioma. **(c)** Respective control. Dot plots are representative of data in Tables S4-S6

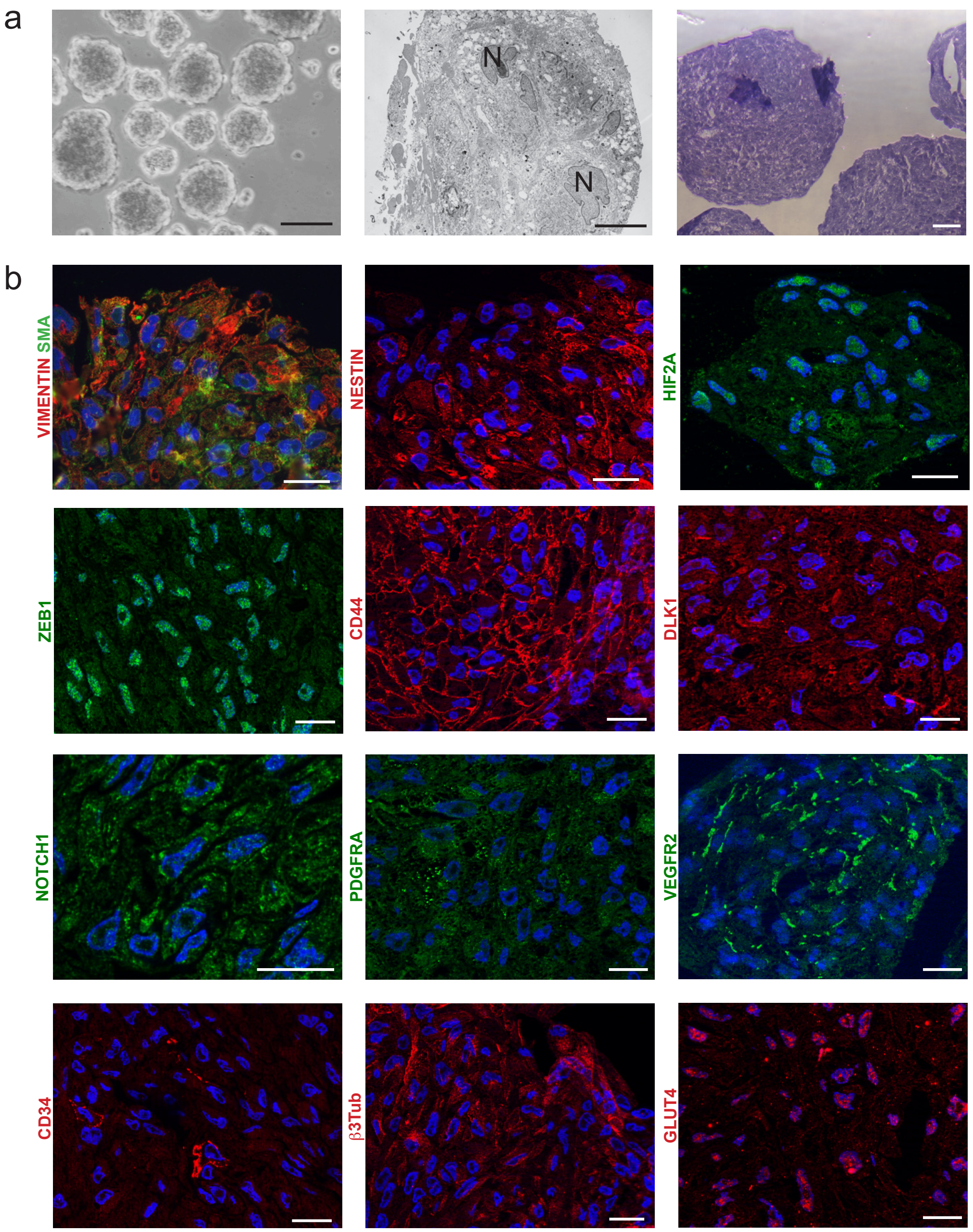

Figure S3

Characteristics of the tumorspheres formed by paraganglioma cells grown under non-adhesive conditions. (a) From left to right: phase contrast micrograph of tumorspheres formed by a representative primary paraganglioma cell culture (PTJ64p, bar = 100 µm); semithin frozen cryo-ultramicrotomy sections of PTJ64p neurospheres stained with toluidine blue (bar = 30 µm); electron micrograph of a small neurosphere showing cohesive cells with vacuolated cytoplasm and large indented nuclei. Bar = 10 µm. **(b)** Optical ApoTome immunofluorescence sections of PTJ64p neurospheres sequentially illustrating: strong positivity for vimentin in the outer cells and for smooth muscle actin (SMA) and nestin in the inner cells; strong nuclear expression of HIF2A and ZEB1; plasma membrane localization of CD44; membrane and cytoplasmic expression of DLK1; mainly cytoplasmic and nuclear expression of NOTCH1, as we previously described in neuroepithelial paraganglioma cells [3]; cytoplasmic and membrane (arrows) expression of PDGFRA; strong VEGFR2 membrane labeling; CD34 plasma membrane staining limited to few individual cells; heterogeneous cytoplasmic expression of β3-tubulin; strong, mainly nuclear GLUT4 expression, as described in undifferentiated embryonic stem cells [4]. Overall, the stem/mesenchymal markers identified in the patient-derived paraganglioma samples are maintained under neurosphere conditions. DAPI is used as a nuclear marker. Bars = 10 µm.

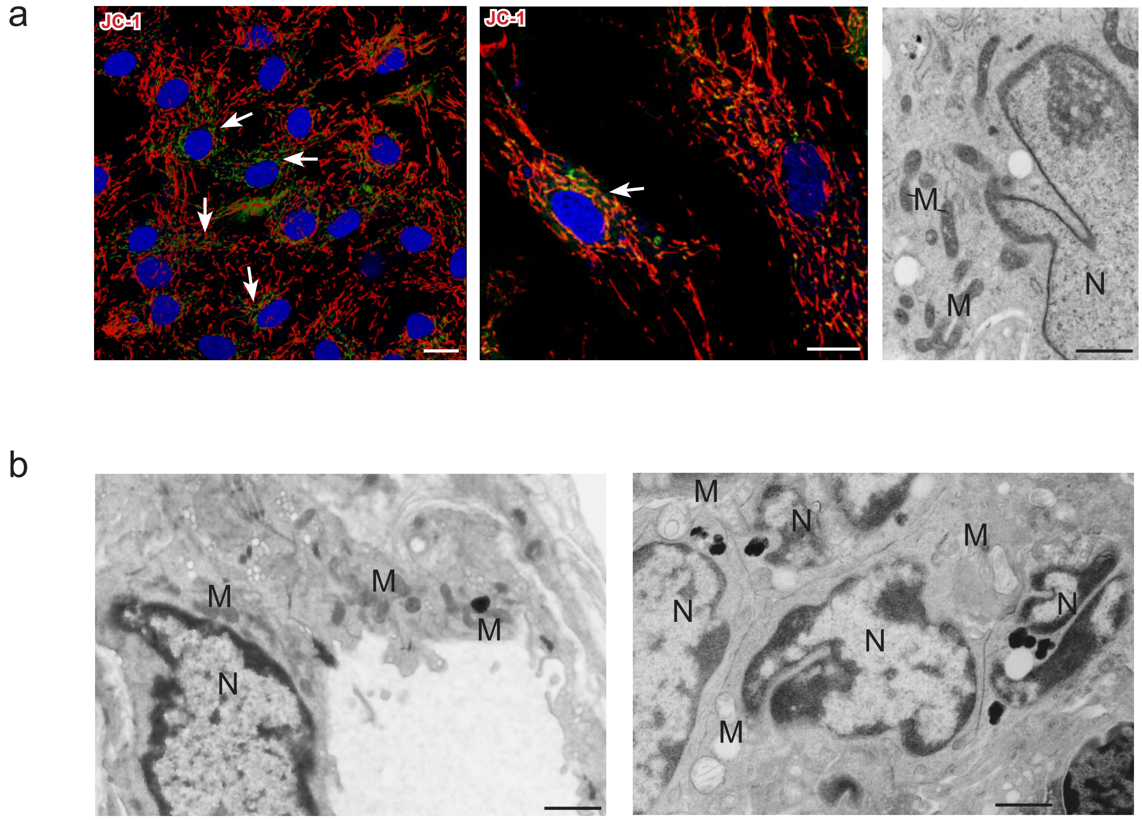

Figure S4

The mitochondria of paraganglioma cells shift from normal to altered morphologies with the transition from *in vitro* to *in vivo* growth. (a) Mitochondrial features of *in vitro* grown PTJ64i cells. Confocal micrographs of JC1-stained PTJ64i cells (left panels) show filamentous mitochondria with prevalently red-fluorescence, indicating high mitochondrial membrane potential. The arrows point to the few green (depolarized) mitochondria (DRAQ5 is used as a nuclear marker). The transmission electron micrograph on the right illustrates ultrastructurally normal tubular mitochondria with well-preserved cristae in a PTJ64i cell (from a neurosphere; M: mitochondria; N: nuclei). Bars = 10 µm (l eft and central images) and 2.5 µm (right image). **(b)** Ultrastructural views of endothelial and neuroepithelial cells from a PTJ64i-derived xenograft. The mitochondria (M) are ultrastructurally well-preserved in the endothelial cell (image on the left), whereas severe mitochondrial swelling with loss of cristae is evident in the neuroepithelial cells (image on the right). N: nuclei; bars = 1 µm

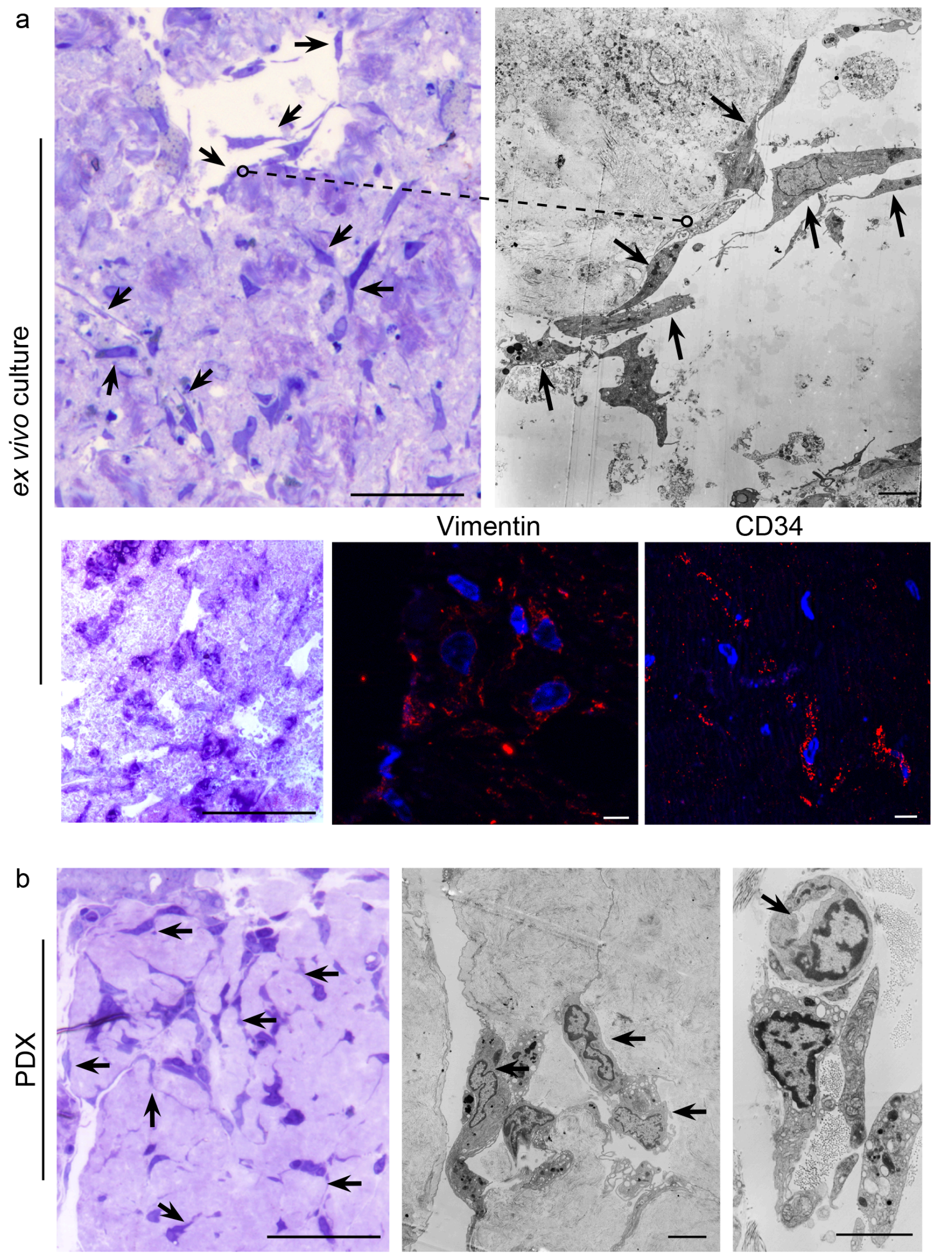

Figure S5

Paraganglioma explants are endogenously recolonized by mesenchymal-like cells similar to those found in the in *vivo* transplanted patient-derived paraganglioma tissue. **(a)** *Ex vivo* cultured paraganglioma explant. Upper panels: semithin section light microscopy and transmission electron microscopy at day 10 of *ex vivo* culture reveal areas endogenously recolonized by elongated, pleomorphic cells with extensive filopodia, forming chains or tubular structures or growing along the necrotic scaffold of pre-existing blood vessels (arrows, dashed lines link comparable optical and electron microscopy areas; bars = 50 µm (image on the left) and 10µm (image on the right). Lower panels: on the left a semithin section light microscopy image illustrates an area not recolonized by endogenous cells, that presents coagulated morphology, dystrophic calcifications (darker staining) and cavities previously occupied by blood vessels (bar = 50µm). The ApoTome immunofluorescence images on the center and on the right show that the endogenous cells recolonizing the necrotic paraganglioma tissue are positive for vimentin and, when forming tubular structures, for CD34 (bars = 10 µm. **(b)** Patient-derived xenograft (at 3 weeks from transplantation). Semithin section microscopy (image on the left) and transmission electron microscopy (central and right images) highlight similar cells within the scaffold of patient-derived *in vivo* transplanted xenografts. Bars = 50 µm (left) and 10 µm (center and right). Toluidine blue is used as a counterstain in all the semithin light microscopy sections

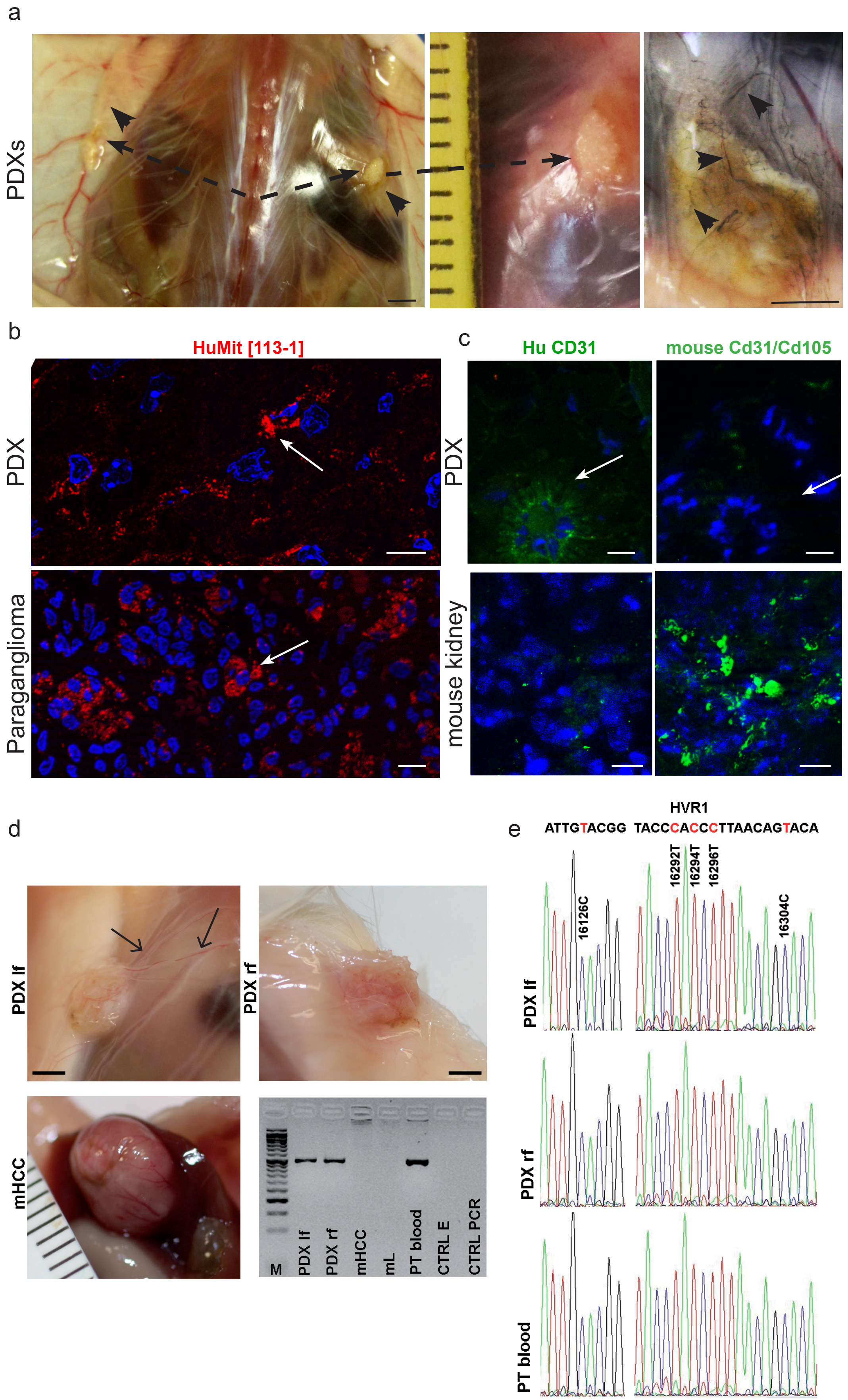
a
PDXs
b
HuMit [113-1]
PDX
Paraganglioma
c
Hu CD31
mouse Cd31/Cd105
PDX
mouse kidney
d
PDX lf
PDX rf
mHCC
M
PDX lf
PDX rf
mHCC
mL
PT blood
CTRL E
CTRL PCR
e
HVR1
ATTGTACGG TACCCACCCTTAACAGTACA
16126C
16292T
16294T
16296T
16304C
PDX lf
PDX rf
PT blood

Figure S6

Gross morphology and human origin of patient-derived paraganglioma xenografts. **(a)** Macroscopic views of bilateral patient-derived paraganglioma xenografts (PDXs) from case PV88 (Table S8). At dissection, 10 months after bilateral transplant, the dorsally exposed subcutaneous and thoracic surfaces of the flanks show whitish nodular growths, ~ 4 mm in diameter, adhering either to the subcutaneous fascia (left flank, arrows, with infiltration of a subcutaneous neurovascular bundle, arrowhead), or to the thoracic cage (right flank, arrow) (bar = 5 mm). A magnification of the right nodule (image on the center) highlights the deflection of an intercostal nerve, pointing to perineural invasion (arrowhead). The image on the right shows a paraganglioma xenograft from case PC98 (Table S8) at 6 months from heterotransplantation, where connections between the neoformed vascular network and the murine vasculature are highlighted with intracardiacally-perfused India ink (arrowheads, bar = 2.5 mm). **(b)** ApoTome immunofluorescence images of a patient-derived xenograft compared to the paraganglioma of origin (semithin sections). The xenograft (PDX) and, below, the paraganglioma of origin (PGL) stain positive with anti-mitochondrial antibody 113-1, which does not recognize rodent mitochondria (arrows). **(c)** ApoTome immunofluorescence images of semithin sections from a patient-derived xenograft (top panels) compared to mouse kidney used as a control for reactivity with murine blood vessels (bottom panels). A vascular cross-section in the PDX stains positive with anti-human CD31 (on the left, arrow), but is not stained with a mix of antibodies to mouse CD31 and CD105 (step section on the right, arrow). Bars =10 µm. **(d)** Paraganglioma xenografts contain patient mt DNA. At dissection, 5 months after subcutaneous transplant, the bilateral xenografts from case PTJ101 present as discrete red-mottled nodules of ~5 mm in maximum diameter (scale bars = 2 mm). The left flank nodule (left PDX) adheres to both the skin flap and the thoracic cage. Several branches of murine intercostal blood vessels and nerves can be seen as they join the left nodule (arrows). The right flank nodule (right PDX) infiltrates the skin flap. The image on the left of the bottom panel illustrates a synchronous ~8 mm-sized liver nodule diagnosed as murine hepatocellular carcinoma (mHCC) by pathological analysis (data not shown), found in the same mouse and used as a control in the mt DNA PCR analysis (next image on the right). PCR analysis targeting a 449 bp fragment of the human mtDNA hypervariable regions I (HVRI) yields specific bands for the left and right PDXs and for the blood of the donor PGL patient (PT blood), but not for the mHCC and for the murine lung control (m-lung). Lanes WL, CTRL-E and CTRL-PCR contain molecular weight ladder, extraction and PCR controls respectively. **(e)** The HVRI sequences from the left and right PDXs (left PDX, right PDX) share the human mt DNA haplotype found in the blood of the PGL patient (PT blood), that differs at 5 positions from the HVRI reference sequence

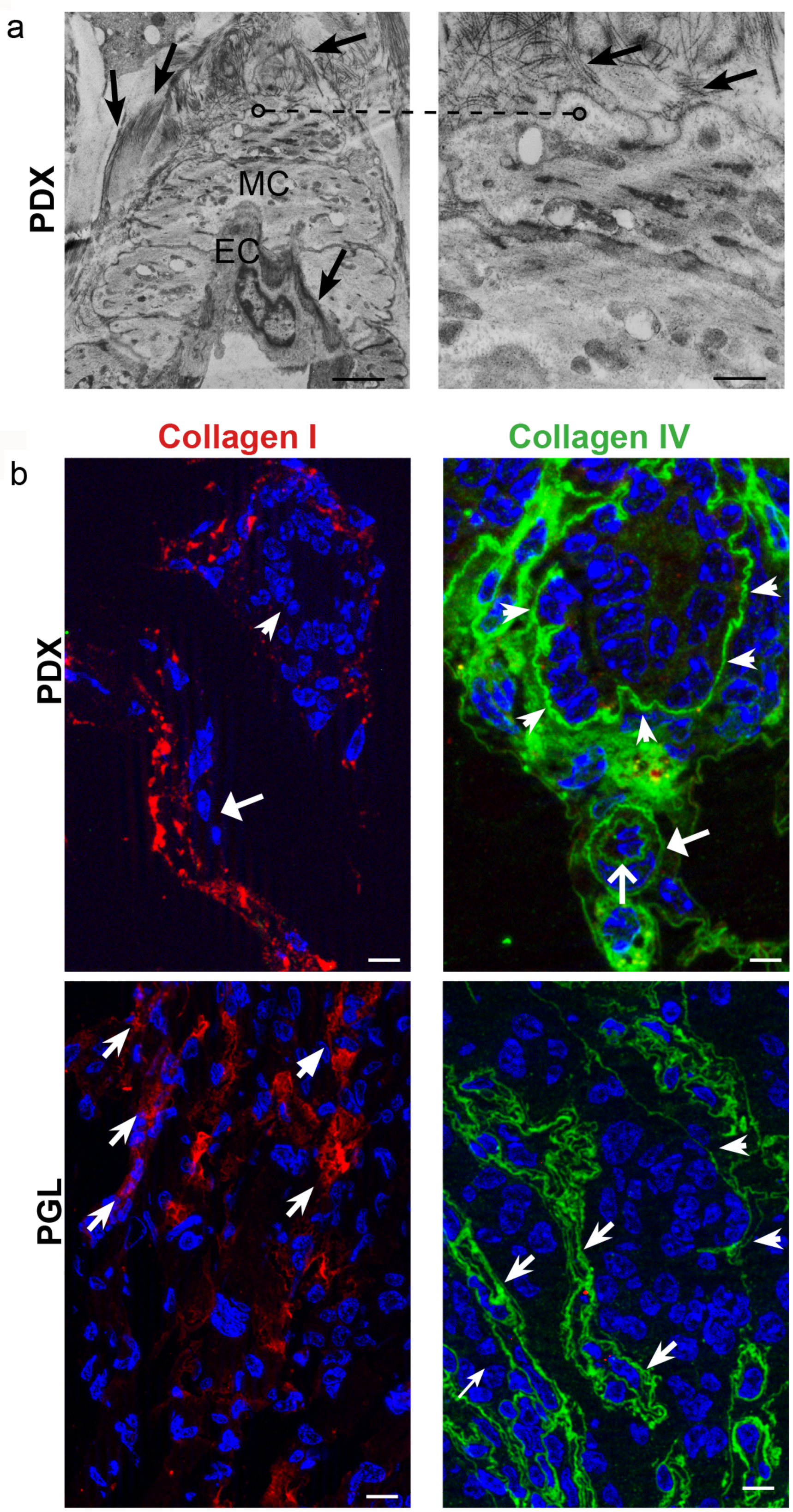

Figure S7
Changes in the collagen matrix accompany paraganglioma xenograft development. **(a)** A tangential ultrastructural section through a neoformed vessel shows the lumen, with an endothelial cell (EC) and thick mural cells (MC). Deposition of fibrillar collagen is visible at the endothelial-mural cell interface and along the outer (ablumenal) membrane of the mural cells on the adventitial side (arrows). The higher magnification view on the right details the release of collagen fibrils next to the outer membrane of a mural cell (arrows). Bars = 10 µm (left) and 1 µm (right). **(b)** Localization of collagens I and IV in developing patient-derived paraganglioma xenografts (PDX) and in paraganglioma. Collagen I (left column, top image) is evident mainly along a neoformed blood vessel (thick arrow), but not along a developing neuroepithelial nest (thin arrow). In a serial section (right column, top image) collagen IV outlines the endothelial-mural cell interface (thin arrow), the adventitial layer of the vascular cross-section (thick arrows) and the periphery of the neuroepithelial nest (thick arrows). In paraganglioma (PGL) collagen 1 is deposited in rather disorganized fashion, mostly along blood vessels (left column, bottom image, thin arrows), whereas collagen IV outlines the basement membranes of the blood vessels (thick arrows) and of the neuroepithelial zellballens (left column, bottom image, thin arrows). DAPI is used as a nuclear marker in the immunofluorescence panels, bars = 10 µm

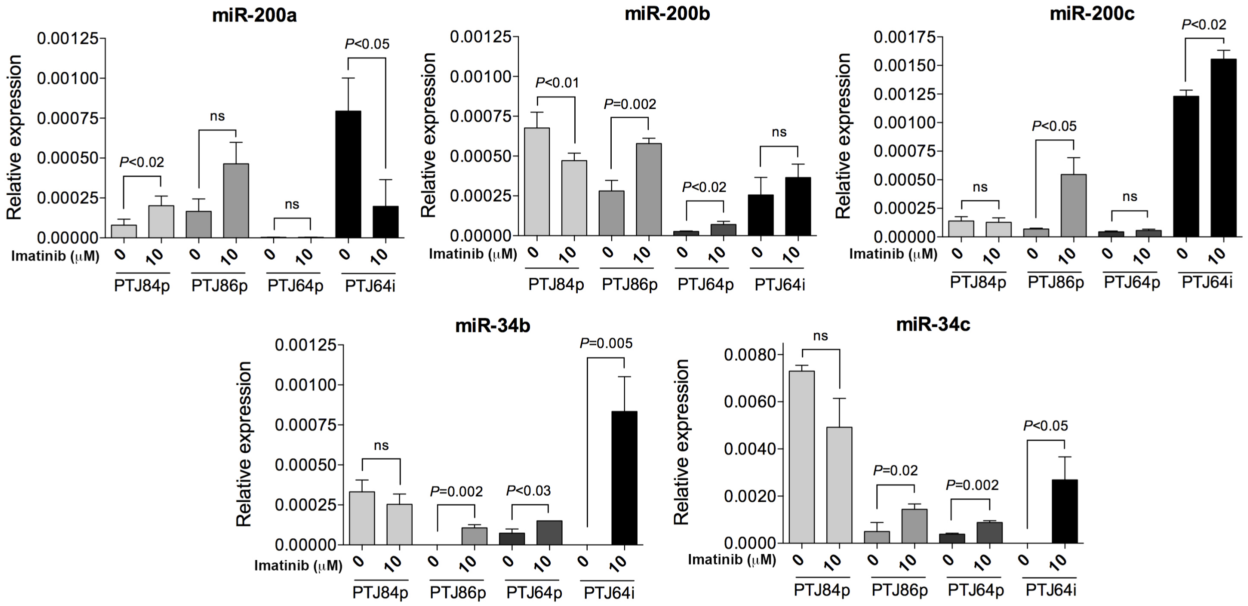

Figure S8

Effects of imatinib on the expression of miR-200a/b/c and miR-34b/c in paraganglioma cells. Real time reverse-transcription PCR of PTJ84p, PTJ86p, PTJ64p and PTJ64i cells at 48 h of treatment with vehicle (0) versus 10 μM imatinib (10) shows significant changes in the relative expression of the miR-200s and of the miR-34s. The expression of the miRNAs is mostly increased, particularly in PTJ86p and PTJ64p cells (carrying germline mutations in *SDHD* and *SDHC*, respectively). Reductions in expression levels were observed for miR-200a in the immortalized cell line PTJ64i and for all miRs (except miR200a) in PTJ84p

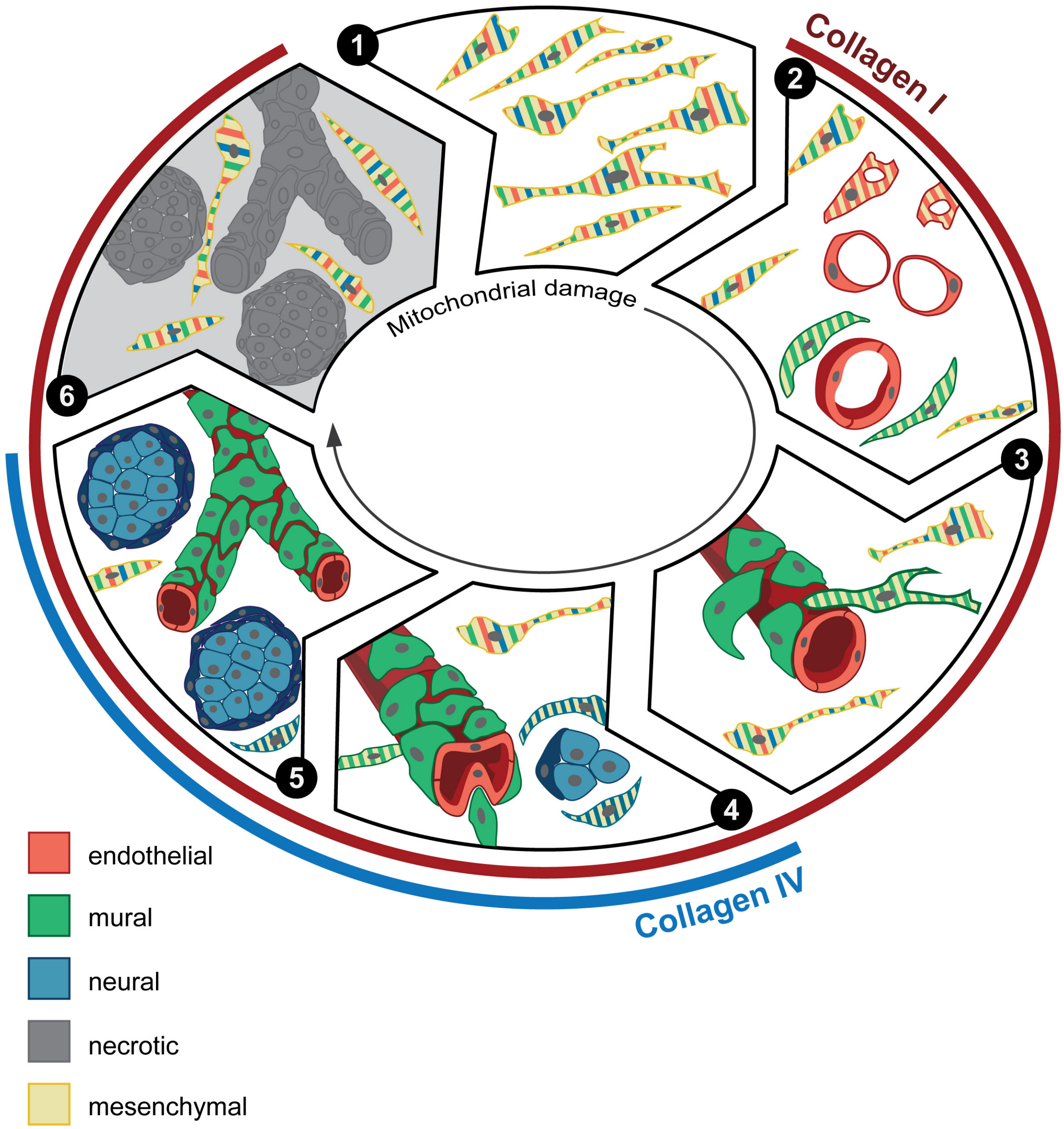

Figure S9

Schematic representation of the phases of PGL development. The thin elongated PGL stem-like cells (phase 1), stabilized and expanded in PGL cell cultures, co-express (stripes) multipotent stem/progenitor cell markers. *In vivo* (phase 2) these cells grow on autonomously synthesized extracellular matrix containing collagen I (external red outline, from phase 1 on), tend to develop intracytoplasmic vacuoles of increasing size (cells with red stripes only), and coalesce (uniformly red cells), giving rise to endothelial tubes (vasculogenesis via cytoplasmic hollowing). The endothelium then recruits adjacent stem-like cells (cells with green stripes only), which, after contact with the abluminal endothelial membranes (phase 3), differentiate into mural cells (uniformly green). Phase 4 outlines two remarkable effects of mural stabilization. First, mural impingement results in intraluminal endothelial intussusceptions, which divide the flow, giving rise to Y-shaped vascular ramifications (intussusceptive angiogenesis, a process detectable only with whole-mount confocal microscopy and/or transmission electron microscopy, as used in

this study [5]). Secondly, vascular stabilization results in perivascular deposition of collagen IV (external blue outline, from phase 3 on), which supports the development of cell clusters with neural phentotype (cells with blue stripes only, then uniformly blue). As shown in phase 5, these clusters develop into "zellballen"-like neuroepithelial nests (uniform light blue) bound by spindle-shaped glia-like cells (uniform dark blue). Phase 6 illustrates the fate of devascularized PGL tissue, where ischemia determines widespread coagulative necrosis (shades of gray). However, mesenchymal-like PGL stem-like cells remain vital and the cycle may start again from such cells, that correspond to those isolated and expanded in culture (phase 1). Notably, while mesenchymal PGL stem-like cells have normal mitochondria, PGL tissue organization is associated with increasing mitochondrial dysfunction (swelling and loss of membrane potential), culminating in the neuroepithelial component

Supplemental references

1. Lachmann R, Lanuti P, Miscia S (2012) OMIP-011: Characterization of circulating endothelial cells (CECs) in peripheral blood. Cytometry A 81: 549-551 Doi 10.1002/cyto.a.22071.
2. Lanuti P, Ciccocioppo F, Bonanni L, Marchisio M, Lachmann R, Tabet N, Pierdomenico L, Santavenere E, Catinella V, Iacone A, Thomas A, Gambi D, Miscia S, Onofrj M, Kern F. Amyloid-specific T-cells differentiate Alzheimer's disease from Lewy body dementia. Neurobiol Aging. 2012 Nov;33(11):2599-611. doi: 10.1016/j.neurobiolaging.2012.01.004.
3. Cama A, Verginelli F, Lotti LV, Napolitano F, Morgano A, D'Orazio A et al (2013) Integrative genetic, epigenetic and pathological analysis of paraganglioma reveals complex dysregulation of NOTCH signaling. Acta Neuropathol 126: 575-594 Doi 10.1007/s00401-013-1165-y.
4. Tonack S, Rolletschek A, Wobus AM, Fischer B, Santos AN (2006) Differential expression of glucose transporter isoforms during embryonic stem cell differentiation. Differentiation 74:499-509.
5. Mentzer SJ, Konerding MA (2014) Intussusceptive angiogenesis: expansion and remodeling of microvascular networks. Angiogenesis 17: 499-509 Doi 10.1007/s10456-014-9428-3
